# Unlocking the therapeutic potential of traditional Chinese medicine in Crohn’s disease: from multi-target mechanisms to clinical promise

**DOI:** 10.3389/fimmu.2026.1719353

**Published:** 2026-10-05

**Authors:** Meng’en Zhou, Yan Chen, Jie Hu, Xiutian Guo, Wenqi Jin

**Affiliations:** Department of Anorectal, Shanghai Municipal Hospital of Traditional Chinese Medicine, Shanghai University of Traditional Chinese Medicine, Shanghai, China

**Keywords:** Crohn’s disease, immune regulation, intestinal barrier, multi-target mechanisms, traditional Chinese medicine

## Abstract

Crohn’s disease (CD) is a chronic, relapsing inflammatory disorder of the gastrointestinal tract with an unclear etiology. Current Western therapies, including 5-aminosalicylates, corticosteroids, biologics, and immunomodulators, often fail to maintain sustained remission and are associated with significant adverse effects and high recurrence rates following drug withdrawal. Traditional Chinese medicine (TCM) has shown potential in alleviating CD symptoms through multi-target and multi-pathway mechanisms. Thus, this review aims to systematically summarize recent advances in understanding the mechanisms by which TCM intervenes in CD, focusing mainly on: regulating immune balance (including modulation of Th1/Th2, Th17/Treg, and M1/M2 macrophage polarization), attenuating inflammatory cascades (via NF-κB, MAPK, PI3K/AKT, and JAK/STAT pathways), reducing oxidative stress, regulating programmed cell death in intestinal epithelial cells (including apoptosis, pyroptosis, ferroptosis, and autophagic cell death), enhancing intestinal mucosal barrier integrity, and promoting restoration of intestinal microbial homeostasis. Furthermore, TCM combined with Western therapies may exert synergistic effects to improve clinical efficacy and reduce relapse risk. Future research could further integrate network pharmacology and multi-omics technologies to elucidate the molecular mechanisms of TCM more deeply, advancing precision therapeutics for CD.

## 1 Introduction

As is well documented, Crohn’s disease (CD) is a chronic inflammatory disorder of the gastrointestinal tract with unclear etiology. Its pathogenesis is associated with genetic susceptibility, immune dysregulation, environmental factors, infections, and alterations in the gut microbiota (1). Its clinical manifestations are heterogeneous and frequently include abdominal pain, diarrhea, abdominal masses, fistula formation, anorectal lesions, and both systemic and extraintestinal symptoms (2). The incidence of CD varies by ethnicity and socioeconomic status. Although rates have historically been high in Europe and North America, they have recently plateaued or declined, whereas emerging industrialized countries in Asia, Africa, and South America continue to report rising incidence (3). Although CD is rarely fatal, its prolonged, relapsing, and refractory course markedly impairs quality of life, imposes considerable psychological and economic burdens on patients, and creates substantial societal costs (4). Current Western medical management relies on 5-aminosalicylates, corticosteroids, monoclonal antibodies, immunomodulators, and surgical interventions. However, these options are limited by suboptimal efficacy, frequent relapse upon drug discontinuation, and significant adverse effects, and fail to comprehensively address the multifaceted nature of CD (5). Traditional Chinese medicine (TCM) has accumulated extensive clinical experience in the management of CD. Indeed, by applying syndrome differentiation and pattern-based therapy, TCM provides individualized treatment plans that effectively alleviate patient symptoms (6).

TCM does not have a precise equivalent to “CD,” but its clinical features allow it to be classified under the TCM entities of “abdominal pain,” “diarrhea,” “intestinal abscess,” and “intestinal obstruction.” The disease arises from congenital weakness, dietary irregularities, external pathogens, and emotional disturbance. It is located mainly in the stomach and intestines, and involves the spleen, liver, kidney, and lung. Its pathogenesis is characterized by spleen deficiency with impaired transportation, dampness-toxin accumulation in the gut, and damage to the intestinal collaterals—root deficiency with branch excess in nature. In terms of syndrome differentiation, CD can be divided into eight patterns: intestinal dampness-heat, turbidity and blood stasis intermingled, heat-toxin and blood stasis, spleen deficiency with dampness retention, cold-heat complex, dual deficiency of qi and blood, spleen-kidney yang deficiency, and yin essence insufficiency. These patterns are further grouped by disease phase: active stage patterns are mainly excess (dampness-heat, turbidity-stasis, heat-toxin), while remission stage patterns are deficiency or mixed (spleen deficiency, cold-heat complex, and various deficiency patterns). This stratification guides formula selection—for example, Huangqin Tang combined with Baitouweng Tang is used for intestinal dampness-heat, Shenling Baizhu Powder for spleen deficiency with dampness retention, and Wumei Wan for cold-heat complex (7, 8).

Although a growing body of mechanistic evidence has emerged regarding the effects of TCM on individual pathways in CD, an integrated synthesis that captures the multi-target, multi-pathway nature of TCM remains limited. Therefore, this review aims to systematically integrate the current mechanistic landscape of TCM in CD, with a particular focus on how TCM modulates the core pathophysiological processes of the disease: restoring immune homeostasis, suppressing inflammatory cascades, protecting intestinal epithelial cells from multiple forms of cell death, reinforcing the intestinal mucosal barrier, and restoring microbial homeostasis. By synthesizing this dispersed evidence, this review constructs a comprehensive mechanistic framework that positions TCM not merely as an adjunctive therapy, but as a multi-targeted therapeutic system with significant potential to address the complex, multifaceted pathogenesis of CD. The mechanisms of action of all TCM compounds, extracts, or active ingredients are summarized in **Tables 1**–**3**.

**Table 1:** Chinese medicine formulations for Crohn’s disease.

Year	Agents	Composition	Mechanisms	References
2010	Daikenchuto	Zingiber officinale Roscoe [Zingiberaceae; Rhizoma Zingiberis Recens], Panax ginseng C.A.Mey. [Araliaceae; Radix Ginseng], Zanthoxylum bungeanum Maxim. [Rutaceae; Pericarpium Zanthoxyli]	TNF-α↓, IFN-γ↓, Adrenomedullin↑	(64)
2020			Eosinophils↓, Visceromotor response↓	(65)
2011			Adrenomedullin↑, Ameliorates colon microvascular dysfunction.	(157)
2011	Baizhu Fuling Decoction	Atractylodes macrocephala Koidz. [Asteraceae; Rhizoma Atractylodis Macrocephalae], Poria cocos (Schw.) Wolf [Polyporaceae; Sclerotium Poriae]	TNF-α↓, IL-1β↓, IL-4↓	(66)
2013			Substance P↓, IL-8↓	(154)
2013			TNF-α↓, IL-1β↓, NF-κB↓	(68)
2013			TNF-α↓, IL-10↑	(69)
2014			TNF-α↓, Glutamine↓	(67)
2014			TNF-α↓, IL-1β↓, IL-6↓, IL-8↓, VIP↓, VIPR1↓, SP↓, NK-1R↓, TLR4↓, NF-κB p65↓, IL-4↑, IL-10↑	(70)
2012	Huangqijiegeng Decoction	Astragalus membranaceus (Fisch.) Bge. [Fabaceae; Radix Astragali], Platycodon grandiflorus (Jacq.) A. DC. [Campanulaceae; Radix Platycodi], Paeonia veitchii Lynch [Paeoniaceae; Radix Paeoniae Rubra], Paeonia lactiflora Pall. [Paeoniaceae; Radix Paeoniae Alba], Citrus reticulata Blanco [Rutaceae; Pericarpium Citri Reticulatae], Forsythia suspensa (Thunb.) Vahl [Oleaceae; Fructus Forsythiae], Coix lacryma-jobi L. var. ma-yuen (Stapf) Keng [Poaceae; Semen Coicis], Bletilla striata (Thunb.) Reichb. f. [Orchidaceae; Rhizoma Bletillae], Scutellaria baicalensis Georgi [Lamiaceae; Radix Scutellariae], Citrus aurantium L. [Rutaceae; Pericarpium Citri Reticulatae], Saposhnikovia divaricata (Turcz.) Schischk. [Umbelliferae; Radix Saposhnikoviae], Diospyros kaki Thunb. [Ebenaceae; Fructus Galla], Glycyrrhiza uralensis Fisch. [Fabaceae; Radix Glycyrrhizae]	VIP↓	(155)
2012			ICAM-1↓	(71)
2013			TGF-β↓	(156)
2012	Huangqihuanglian Decoction	Astragalus membranaceus (Fisch.) Bge. [Fabaceae; Radix Astragali],Coptis chinensis Franch. [Ranunculaceae; Rhizoma Coptidis], Aucklandia lappa Decne. [Asteraceae; Radix Aucklandiae], Paeonia veitchii Lynch [Paeoniaceae; Radix Paeoniae Rubra], Paeonia lactiflora Pall. [Paeoniaceae; Radix Paeoniae Alba], Glycyrrhiza uralensis Fisch. [Fabaceae; Radix Glycyrrhizae], Panax notoginseng (Burk.) F.H.Chen [Araliaceae; Radix Notoginseng], Diospyros kaki Thunb. [Ebenaceae; Fructus Galla],Atractylodes macrocephala Koidz. [Asteraceae; Rhizoma Atractylodis Macrocephalae], Areca catechu L. [Arecaceae; Semen Arecae], Sanguisorba officinalis L. [Rosaceae; Radix Sanguisorbae], Taraxacum mongolicum Hand.-Mazz. [Asteraceae; Herba Taraxaci],Coix lacryma-jobi L. var. ma-yuen (Stapf) Keng [Poaceae; Semen Coicis]	VIP↓	(155)
2012			ICAM-1↓	(71)
2012			TGF-β↓	(156)
2014	Xianglian Pill	Coptis chinensis Franch. [Ranunculaceae; Rhizoma Coptidis], Evodia rutaecarpa (Juss.) Benth. [Rutaceae; Fructus Evodiae], Aucklandia lappa Decne. [Asteraceae; Radix Aucklandiae], Prunus mume Sieb. et Zucc. [Rosaceae; Fructus Mume]	TNF-α↓, IL-1β↓, IL-4↓	(67)
2014	Baizhu Fuling Decoction combined with Xianglian Pill	Atractylodes macrocephala Koidz. [Asteraceae; Rhizoma Atractylodis Macrocephalae], Poria cocos (Schw.) Wolf [Polyporaceae; Sclerotium Poriae], Coptis chinensis Franch. [Ranunculaceae; Rhizoma Coptidis], Evodia rutaecarpa (Juss.) Benth. [Rutaceae; Fructus Evodiae], Aucklandia lappa Decne. [Asteraceae; Radix Aucklandiae], Prunus mume Sieb. et Zucc. [Rosaceae; Fructus Mume]	TNF-α↓, IL-1β↓, IL-4↓, Glutamine↓	(67)
2015	Compound extract of Flos Abelmoschi Manihot and Fructus Mume	Abelmoschus manihot (L.) Medic. [Malvaceae; Flos Abelmoschi], Prunus mume (Sieb. et Zucc.) [Rosaceae; Fructus Mume]	TNF-α↓, IFN-γ↓	(72)
2016	Spleen-invigorating Prescription	Psoralea corylifolia L. [Fabaceae; Fructus Psoraleae], Pulsatilla chinensis (Bunge) Regel [Ranunculaceae; Radix Pulsatillae], Portulaca oleracea L. [Portulacaceae; Herba Portulacae], Poria cocos (Schw.) Wolf [Polyporaceae; Sclerotium Poriae], Atractylodes macrocephala Koidz. [Asteraceae; Rhizoma Atractylodis Macrocephalae], Agastache rugosa (Fisch. ex Benth.) Kuntze [Lamiaceae; Herba Agastachis]	CCL20↓, CCR6↓, NF-κB p65↓, IL-23↓	(73)
2017			IFN-γ↓, IL-17↓, RORγt↓, FoxP3↑	(48)
2016	Huangqin Tang Granules	Coptis chinensis Franch. [Ranunculaceae; Rhizoma Coptidis], Zingiber officinale Roscoe [Zingiberaceae; Rhizoma Zingiberis Recens], Aucklandia lappa Decne. [Asteraceae; Radix Aucklandiae], Magnolia officinalis Rehd. et Wils. [Magnoliaceae; Cortex Magnoliae Officinalis]	TH17↓, Treg↑, IL-17↓, IL-6↓, IL-10↑	(145)
2016	Kuijiekang	Atractylodes macrocephala Koidz. [Asteraceae; Rhizoma Atractylodis Macrocephalae], Poria cocos (Schw.) Wolf [Polyporaceae; Sclerotium Poriae], Paeonia lactiflora Pall. [Paeoniaceae; Radix Paeoniae Alba], Citrus reticulata Blanco [Rutaceae; Pericarpium Citri Reticulatae], Saposhnikovia divaricata (Turcz.) Schischk. [Apiaceae; Radix Saposhnikoviae], Platycodon grandiflorus (Jacq.) A. DC. [Campanulaceae; Radix Platycodi], Panax notoginseng (Burk.) F.H.Chen [Araliaceae; Radix Notoginseng], Sophora japonica L. [Fabaceae; Flos Sophorae Immaturus], Sanguisorba officinalis L. [Rosaceae; Radix Sanguisorbae], Glycyrrhiza uralensis Fisch. [Fabaceae; Radix Glycyrrhizae]	IFN-γ↓, IL-1↓, Glutamine↑, IL-4↑, IL-10↑	(74)
2017	Shenling Baizhu Powder	Atractylodes macrocephala Koidz. [Asteraceae; Rhizoma Atractylodis Macrocephalae], Dioscorea opposita Thunb. [Dioscoreaceae; Rhizoma Dioscoreae], Nelumbo nucifera Gaertn. [Nelumbonaceae; Semen Nelumbinis], Poria cocos (Schw.) Wolf [Polyporaceae; Sclerotium Poriae], Coix lacryma-jobi L. var. ma-yuen (Stapf) Keng [Poaceae; Semen Coicis], Platycodon grandiflorus (Jacq.) A. DC. [Campanulaceae; Radix Platycodi], Amomum villosum Lour. [Zingiberaceae; Fructus Amomi], Lablab purpureus (L.) Sweet [Fabaceae; Semen Lablab], Panax ginseng C.A.Mey. [Araliaceae; Radix Ginseng], Glycyrrhiza uralensis Fisch. [Fabaceae; Radix Glycyrrhizae], Ziziphus jujuba Mill. [Rhamnaceae; Fructus Jujubae]	IL-1β↓, IL-4↑, IL-10↑	(75)
2021			miR-21↓, miR-150↓, Claudin-1↑, miR-200b↑	(126)
2023			IL-1β↓, IL-17C↓, IL-1↓, IL-10↑, IL-4↑	(76)
2023			Occludin↑, ZO-1↑, E-cadherin↑	(127)
2023			TNF-α↓, IL-1↓, IL-6↓, p-p38↓, p-p65↓	(77)
2023			IL-17A↓, TNF-α↓, CD4+T↓, CD8+T↑	(152)
2024			p-PI3K/PI3K↓, p-Akt/Akt↓	(78)
2018	Tou Nong San	Astragalus membranaceus (Fisch.) Bge. [Fabaceae; Radix Astragali], Angelica sinensis (Oliv.) Diels [Apiaceae; Radix Angelicae Sinensis], Ligusticum chuanxiong Hort. [Apiaceae; Rhizoma Chuanxiong], Gleditsia sinensis Lam. [Fabaceae; Spina Gleditsiae]	TNF-α↓, IL-6↓, IL-1β↓, IL-17↓, NF-κB p65↓, p-IKKβ↓, IκBα↑	(79)
2019	Chaihu Guizhi decoction	Paeonia lactiflora Pall. [Paeoniaceae; Radix Paeoniae Alba], Codonopsis pilosula (Franch.) Nannf. [Campanulaceae; Radix Codonopsis], Bupleurum chinense DC. [Apiaceae; Radix Bupleuri], Cinnamomum cassia Presl [Lauraceae; Ramulus Cinnamomi], Astragalus membranaceus (Fisch.) Bge. [Fabaceae; Radix Astragali], Glycyrrhiza uralensis Fisch. [Fabaceae; Radix Glycyrrhizae], Ziziphus jujuba Mill. [Rhamnaceae; Fructus Jujubae], Zingiber officinale Roscoe [Zingiberaceae; Rhizoma Zingiberis Recens]	CD3↓, CD4↓, CD4/CD8↓, CD8↑	(146)
2019	Chaihu Shugan Powder combined with Shenling Baizhu Powder	Bupleurum chinense DC. [Apiaceae; Radix Bupleuri], Codonopsis pilosula (Franch.) Nannf. [Campanulaceae; Radix Codonopsis], Poria cocos (Schw.) Wolf [Polyporaceae; Sclerotium Poriae], Dioscorea opposita Thunb. [Dioscoreaceae; Rhizoma Dioscoreae], Lablab purpureus (L.) Sweet [Fabaceae; Semen Lablab], Atractylodes macrocephala Koidz. [Asteraceae; Rhizoma Atractylodis Macrocephalae], Citrus aurantium L. [Rutaceae; Pericarpium Citri Reticulatae], Nelumbo nucifera Gaertn. [Nelumbonaceae; Semen Nelumbinis], Amomum villosum Lour. [Zingiberaceae; Fructus Amomi], Coix lacryma-jobi L. var. ma-yuen (Stapf) Keng [Poaceae; Semen Coicis], Cyperus rotundus L. [Cyperaceae; Rhizoma Cyperi], Citrus reticulata Blanco [Rutaceae; Pericarpium Citri Reticulatae], Paeonia lactiflora Pall. [Paeoniaceae; Radix Paeoniae Alba], Platycodon grandiflorus (Jacq.) A. DC. [Campanulaceae; Radix Platycodi], Glycyrrhiza uralensis Fisch. [Fabaceae; Radix Glycyrrhizae]	Th17↑,Treg(CD4+ CD25+)↑,IL-10↑,TNF-α↓, IL-17↓, IL-6↓	(147)
2019	Modified Taohua Decoction combined with Buzhong Yiqi Decoction	Codonopsis pilosula (Franch.) Nannf. [Campanulaceae; Radix Codonopsis], Astragalus membranaceus (Fisch.) Bge. [Fabaceae; Radix Astragali], Atractylodes macrocephala Koidz. [Asteraceae; Rhizoma Atractylodis Macrocephalae], Angelica sinensis (Oliv.) Diels [Apiaceae; Radix Angelicae Sinensis], Citrus reticulata Blanco [Rutaceae; Pericarpium Citri Reticulatae], Bupleurum chinense DC. [Apiaceae; Radix Bupleuri], Petalostigma chinense (Lour.) Merr. [Euphorbiaceae; Lipstick Tree Resin (Resina Draconis)], Zingiber officinale Roscoe [Zingiberaceae; Rhizoma Zingiberis Recens], Glycyrrhiza uralensis Fisch. [Fabaceae; Radix Glycyrrhizae Preparata], Smilax glabra Roxb. [Smilacaceae; Rhizoma Smilacis Glabrae], Geranium japonicum Thunb. [Geraniaceae; Herba Geranii Japonici], Bletilla striata (Thunb.) Reichb. f. [Orchidaceae; Rhizoma Bletillae], Cyathula officinalis DC. [Amaranthaceae; Radix Cyathulae]	INF-γ↓, TNF-α↓, IL-2↓, IL-17↓	(148)
2020	Modified Lizhong Decoction	Zingiber officinale Roscoe [Zingiberaceae; Rhizoma Zingiberis Recens], Codonopsis pilosula (Franch.) Nannf. [Campanulaceae; Radix Codonopsis],Atractylodes macrocephala Koidz. [Asteraceae; Rhizoma Atractylodis Macrocephalae], Dioscorea opposita Thunb. [Dioscoreaceae; Rhizoma Dioscoreae], Lablab purpureus (L.) Sweet [Fabaceae; Semen Lablab], Foeniculum vulgare Mill. [Apiaceae; Fructus Foeniculi], Terminalia chebula Retz. [Combretaceae; Pericarpium Terminaliae Chebulae], Alpinia oxyphylla Miq. [Zingiberaceae; Fructus Alpiniae Oxyphyllae], Atractylodes lancea (Thunb.) DC. [Asteraceae; Rhizoma Atractylodis Lanceae] Coix lacryma-jobi L. var. ma-yuen (Stapf) Keng [Poaceae; Semen Coicis], Aucklandia lappa Decne. [Asteraceae; Radix Aucklandiae], Corydalis yanhusuo W.T.Wang [Papaveraceae; Rhizoma Corydalis], Panax notoginseng (Burk.) F.H.Chen [Araliaceae; Radix Notoginseng], Bletilla striata (Thunb.) Reichb. f. [Orchidaceae; Rhizoma Bletillae], Paeonia lactiflora Pall. [Paeoniaceae; Radix Paeoniae Alba], Glycyrrhiza uralensis Fisch. [Fabaceae; Radix Glycyrrhizae Preparata]	Th17↓, Th17/Treg ↓, Treg↑, IL-6↓, IL-35↑	(149)
2022	Fuzheng Huoxue Decoction	Salvia miltiorrhiza Bge. [Lamiaceae; Radix et Rhizoma Salviae Miltiorrhizae], Astragalus membranaceus (Fisch.) Bge. [Fabaceae; Radix Astragali], Panax notoginseng (Burk.) F.H.Chen [Araliaceae; Radix Notoginseng], Poria cocos (Schw.) Wolf [Polyporaceae; Sclerotium Poriae], Angelica sinensis (Oliv.) Diels [Apiaceae; Radix Angelicae Sinensis], Dioscorea opposita Thunb. [Dioscoreaceae; Rhizoma Dioscoreae], Ligusticum chuanxiong Hort. [Apiaceae; Rhizoma Ligustici Chuanxiong], Magnolia officinalis Rehd. et Wils. [Magnoliaceae; Cortex Magnoliae Officinalis], Paeonia veitchii Lynch [Paeoniaceae; Radix Paeoniae Rubra], Lindera aggregata (Sims) Kosterm. [Lauraceae; Radix Linderae], Citrus aurantium L. [Rutaceae; Pericarpium Citri Reticulatae], Corydalis yanhusuo W.T.Wang [Papaveraceae; Rhizoma Corydalis], Crataegus cuneata Sieb. et Zucc. [Rosaceae; Fructus Crataegi], Glycyrrhiza uralensis Fisch. [Fabaceae; Radix Glycyrrhizae]	TNF-α↓, Th1/Th2↓, IL-10↑	(150)
2022	Xue-Jie-San	Dracaena cochinchinensis (Lour.) S.C.Chen [Dracaenaceae; Dracaena], Commiphora myrrha Engl. [Burseraceae; Myrrha]	IL-8↓, IL-1β↓, TNF-α↓, Beclin1↓, LC3B↓, NLRP3↓, AIM2↓, ASC↓, Caspase-1↓, p-mTOR↑	(80)
2022			IL-6↓, TNF-α↓, IL-17↓, 4-HNE↓, Notch1↓, NICD1↓, PTGS2↓, Nrf2↑, GPX4↑	(81)
2024			IL-1β↓, IL-6↓, TNF-α↓, CCL2↓, CXCL8↓, TLR4↓, Myd88↓, p-p65↓, ICAM-1↓	(82)
2023	Buzhong Yiqi Decoction	Astragalus membranaceus (Fisch.) Bge. [Fabaceae; Radix Astragali], Bupleurum chinense DC. [Apiaceae; Radix Bupleuri], Zingiber officinale Roscoe [Zingiberaceae; Rhizoma Zingiberis Recens], Atractylodes macrocephala Koidz. [Asteraceae; Rhizoma Atractylodis Macrocephalae], Angelica sinensis (Oliv.) Diels [Apiaceae; Radix Angelicae Sinensis], Glycyrrhiza uralensis Fisch. [Fabaceae; Radix Glycyrrhizae], Citrus reticulata Blanco [Rutaceae; Pericarpium Citri Reticulatae], Cimicifuga heracleifolia Kom. [Ranunculaceae; Rhizoma Cimicifugae], Panax ginseng C.A.Mey. [Araliaceae; Radix Ginseng], Ziziphus jujuba Mill. [Rhamnaceae; Fructus Jujubae]	CD3+↓, CD4+↓, CD4+/CD25+ Treg↑	(151)
2023	Huanghou zhixie dropping pill	Coptis chinensis Franch. [Ranunculaceae; Rhizoma Coptidis], Zingiber officinale Roscoe [Zingiberaceae; Rhizoma Zingiberis Recens], Aucklandia lappa Decne. [Asteraceae; Radix Aucklandiae], Magnolia officinalis Rehd. et Wils. [Magnoliaceae; Cortex Magnoliae Officinalis]	IL-6↓, TNF-α↓, IL-1β↓, NF-κB p65↓, p-IKBα↓, p-PI3K↓, p-Akt↓, MPO↓, IFN-γ↓, IL-2↓	(83)
2023	Qinghua Xiaoyong Formula	Scutellaria baicalensis Georgi [Lamiaceae; Radix Scutellariae],Coptis chinensis Franch. [Ranunculaceae; Rhizoma Coptidis],Phellodendron chinense Schneid. [Rutaceae; Cortex Phellodendri]	TNF-α↓, IL-6↓, IL-1β↓, p-PI3K/PI3K↓, p-AKT/AKT↓, SOD↑, GSH↑, MDA↓	(84)
2024	Modified Banxia Xiexin Decoction	Coptis chinensis Franch. [Ranunculaceae; Rhizoma Coptidis], Pinellia ternata (Thunb.) Breit. [Araceae; Rhizoma Pinelliae], Scutellaria baicalensis Georgi [Lamiaceae; Radix Scutellariae], Zingiber officinale Roscoe [Zingiberaceae; Rhizoma Zingiberis Recens], Evodia rutaecarpa (Juss.) Benth. [Rutaceae; Fructus Evodiae], Codonopsis pilosula (Franch.) Nannf. [Campanulaceae; Radix Codonopsis], Glycyrrhiza uralensis Fisch. [Fabaceae; Radix Glycyrrhizae], Ziziphus jujuba Mill. [Rhamnaceae; Fructus Jujubae], Magnolia officinalis Rehd. et Wils. [Magnoliaceae; Cortex Magnoliae Officinalis], Citrus reticulata Blanco [Rutaceae; Pericarpium Citri Reticulatae], Bupleurum chinense DC. [Apiaceae; Radix Bupleuri], Paeonia lactiflora Pall. [Paeoniaceae; Radix Paeoniae Alba]	IL-6↓, TNF-α↓, IL-17↓, Bifidobacterium ↑, Lactobacillus ↑, Faecalibacterium prausnitzii ↑, Proteobacteria ↓, Klebsiella ↓, Enterococcus ↓	(153)
2025	Changyanning tablet	Euphorbia humifusa Willd. [Euphorbiaceae; Herba Euphorbiae Humifusae],Hedyotis chrysotricha (Palib.) Merr. [Rubiaceae; Herba Hedyotidis Chrysotrichae], Cinnamomum camphora (L.) J.Presl [Lauraceae; Radix Cinnamomi Camphorae], Elsholtzia ciliata (Thunb.) Hyl. [Lamiaceae; Herba Elsholtziae], Liquidambar formosana Hance [Altingiaceae; Folium Liquidambaris Formosanae],	TNF-α↓, IL-6↓, GSH↑, SLC7A11↑, GPX4↑, MDA↓, Occludin↑, Claudin-1↑	(85)
2025	Xiangsha Liujunzi Wan	Poria cocos (Schw.) Wolf [Polyporaceae; Sclerotium Poriae], Glycyrrhiza uralensis Fisch. [Fabaceae; Radix Glycyrrhizae], Atractylodes macrocephala Koidz. [Asteraceae; Rhizoma Atractylodis Macrocephalae], Panax ginseng C.A.Mey. [Araliaceae; Radix Ginseng], Citrus reticulata Blanco [Rutaceae; Pericarpium Citri Reticulatae], Arum ternatum Thunb. [Araceae; Rhizoma Arisaematis], Amomum aurantiacum H. T. Tsai et S. W. Zhao [Zingiberaceae; Fructus Amomi Aurantii]	TNF-α↓, IL-6↓, IL-1β↓, p-PI3K↓, p-AKT↓, p-JNK↓, p-p38↓, ZO-1↑, Occludin↑	(86)

↑, increase; ↓, decrease; ACSL4, acyl-CoA synthetase long-chain family member 4; AIM2, absent in melanoma 2; ASC, apoptosis-associated speck-like protein containing a CARD; CCL20, C-C motif chemokine ligand 20; CCR6, C-C chemokine receptor type 6; CDAI, Crohn’s Disease Activity Index; COX-2, cyclooxygenase-2; FoxP3, forkhead box P3; GPX4, glutathione peroxidase 4; GSH, glutathione; ICAM-1, intercellular adhesion molecule-1; IEC, intestinal epithelial cell; I-FABP, intestinal fatty-acid-binding protein; IL, interleukin; iNOS, inducible nitric oxide synthase; MDA, malondialdehyde; MLCK, myosin light chain kinase; MPO, myeloperoxidase; MUC2, mucin 2; MyD88, myeloid differentiation primary response 88; NF-κB, nuclear factor-kappa B; NICD1, notch intracellular domain 1; NK-1R, neurokinin-1 receptor; Nrf2, nuclear factor erythroid 2-related factor 2; PTGS2, prostaglandin-endoperoxide synthase 2; RORγt, RAR-related orphan receptor gamma t; ROS, reactive oxygen species; SLC7A11, solute carrier family 7 member 11; SOD, superoxide dismutase; SP, substance P; TEER, transepithelial electrical resistance; TGF-β, transforming growth factor-beta; TLR4, Toll-like receptor 4; TNF-α, tumor necrosis factor-alpha; VIP, vasoactive intestinal peptide; VIPR1, vasoactive intestinal peptide receptor 1; ZO-1, zonula occludens-1; 4-HNE, 4-hydroxynonenal.

**Table 2:** Traditional Chinese medicine herbal extracts for Crohn’s disease.

Year	Agents	Composition	Mechanisms	References
2012	Pomegranate extract and ellagic acid	Punica granatum L. [Lythraceae; Fructus Punicae]	MPO↓, TNF-α↓, COX-2↓, iNOS↓, p-JUNK↓, p-p38↓, p-ERK1/2↓, Iκβ-α↓, NF-κB p65↓	(87)
2018	Rosa laevigata Fructus extract	Rosa laevigata Michx. [Rosaceae; Fructus Rosae Laevigatae]	TNF-α↓, IFN-γ↓	(88)
2019	Brucea javanica oil	Brucea javanica (L.) Merr. [Simaroubaceae; Fructus Bruceae]	MPO↓, TNF-α↓, IL-1β↓, IL-6↓, IL-17↓, IL-23↓, IFN-γ↓, IL-4↑, IL-10↑, TGF-β↑, MMP-1↓, MMP-3↓, RAGE↓, PPAR-γ↑, TLR4↓, MyD88↓, p-p65↓, TRAF-6↓	(89)
2022	Mume Fructus	Prunus mume (Sieb. et Zucc.) [Rosaceae; Mume Fructus]	IL-1β↓, IL-6↓, IL-17↓, TGF-β↓, LPS↓, ZO-1↑, Occludin↑, Acetic↓, Propionic↓, Butyric↓, Total acids↓, Enhance intestinal microbial diversity.	(90)
2022			MPO↓, NO↓, IL-1β↓, IL-6↓, IL-17↓, IL-10↑, Modulate intestinal short-chain fatty acid levels.	(91)

↑, increase; ↓, decrease; COX-2, cyclooxygenase-2; IFN-γ, interferon-gamma; IL, interleukin; iNOS, inducible nitric oxide synthase; LPS, lipopolysaccharide; MMP, matrix metalloproteinase; MPO, myeloperoxidase; MyD88, myeloid differentiation primary response 88; NF-κB, nuclear factor-kappa B; PPAR-γ, peroxisome proliferator-activated receptor gamma; RAGE, receptor for advanced glycation end products; TGF-β, transforming growth factor-beta; TLR4, Toll-like receptor 4; TNF-α, tumor necrosis factor-alpha; TRAF-6, TNF receptor-associated factor 6; ZO-1, zonula occludens-1.

**Table 3:** Active ingredients from medicinal herbs for Crohn’s disease.

Year	Agents	Mechanisms	References
2011	Triptolide	MPO↓, IL-6↓, p-STAT3↓, gp130↓, RORγt↓, Bcl-2↓, Bcl-xl↓, IL-17↓	(49)
2011		MPO↓, TLR2↓, TLR4↓, MyD88↓, NF-κB p65↓, TNF-α↓, INF-γ↓, MHCII↓, CD86↓	(92)
2011	Ellagic acid	MPO↓, COX-2↓, iNOS↓, p-JUNK↓, p-p38↓, p-ERK1/2↓, Iκβ-α↓, NF-κB p65↓	(93)
2012	Artesunate	IFN-γ↓, IL-17↓, TNF-α↓, MPO↓, NF-κB p65↓, p-IκB-α↓	(50)
2013	Tripterygium glycosides	Smad3↓, Smad4↓, TGF-β1↓, p-Smad2/3↓, Smad7↑	(94)
2014		Foxp3+ Tregs↑, IL-10↑, Foxp3↑, TNF-α↓	(51)
2015	Berberine	IFN-γ↓, IL-17↓, IL-6↓, IL-1β↓, TNF-α↓, MPO↓, IL-10↑, IL-22↑, IgA↑, IRF4/IRF5↑, T-bet↓, RORt↓, TH-1↓, Th-17↓, p-STAT1↓, p-STAT3↓, NF-κB p-65↓, Restore Treg homeostasis.	(52)
2017	Baicalein	MPO↓, iNOS↓, ICAM-1↓, MCP-1↓, COX-2↓, TNF-α↓, IL-1β↓, p-p65↓, p-IκBα↓, p-p38↓, TLR4↓, MyD88↓, p-IRAK-1↓, COX-2↓, IL-1α↓, IL-1β↓, NO↓, p-ERK1/2↓, p-JNK↓, NLRP3↓, ASC↓, Caspase-1↓, IL-1β↓	(95)
2017	Quercitrin	iNOS↓, COX2↓, NOX1↓, TNFα↓, IL1β↓	(96)
2019	Total flavone of Abelmoschus Manihot	TNF-α↓, IFN-γ↓, IL-6↓, IL-1β↓, IL-12↓, IL-17↓, p-IKKα/β/γ↓, IκB↓, p-p65↓, p100↑, p52↑, LC3-II/LC3-II↑, Beclin-1↑, p62↓	(97)
2019		TNF-α↓, IFN-γ↓, IL-6↓, IL-1β↓, IL‑12↓, IL‑17↓, MPO↓, p-IKK↓, IκBα↓, p100↑, p52↑, p-p65↓, p-JUN↓, p-38↓	(98)
2019	Procyanidin B2	TNF-α↓, IL-1β↓, p-PI3K, p-AKT↓, IL-10↑, FITC-dextran↓, Claudin-1↑, ZO-1↑. Significantly attenuated intestinal bacterial translocation to mesenteric lymph nodes.	(99)
2019	Clematichinenoside AR	IL-1β↓, TNF-α↓, IL-17A↓, Bcl-2↑, Cleaved caspase-3↓, Bax↓, Tregs↑, Th17↓, p-PI3K↓, p-Akt↓, Occludin↑, ZO-1↑, Significantly attenuated intestinal bacterial translocation to mesenteric lymph nodes, liver, and spleen.	(53)
2020	Tetramethylpyrazine	IL-2↓, IL-12↓, IFN-γ↓, TNF-α↓, ICAM-1↓, iNOS↓, P-selectin↓	(100)
2020	Loureirin B	IL-1↓, IL-1β↓, IL-6↓, TNF-α↓, TGF-β↓, INOS↓, MPO↓, MDA↓, Snai↓↓, N-cadherin↓, Vimentin↓, Bax↓, Cleaved caspase-3↓, Cleaved caspase-9↓, IL-6↓, p-STAT3↓, p-IKB-α↑, p-p65, IL-10↑, SIgA↑, E-cadherin↑, Bcl-2↑	(101)
2022	Ruscogenins	Nrf2↑, NQO1↑, TNF-α↓, IFN-γ↓, G-CSF↓, RANTES↓, MCP1↓, Bax↓, Cleaved caspase-3↓, Bcl-2↑, FITC-dextran↓, TEER↑, Occludin↑, ZO-1↑, Significantly attenuated intestinal bacterial translocation to mesenteric lymph nodes, liver.	(102)
2023	Pachymic acid	TNF-α↓, IL-6↓, IL-1β↓, p-PI3K↓, p-AKT↓, FITC-dextran↓, I-FABP↓, TEER↑	(103)
2023	Phillygenin	TNF-α↓, IL-6↓, IFN-γ↓, IL-17↓, IL-17 RA↓, NF-κB activor1↓	(104)
2023	Eriocalyxin B	IL-6↓, TNF-α↓, IL-1β↓, iNOS↓, CD86↓, p-JAK2/JAK2↓, p-STAT1/STAT1↓	(54)
2023	Diosmetin	TNF-α↓, IFN-γ↓, IL-17A↓, Th1/Th2↓, Th17/Treg↓, p-PI3K↓, p-AKT↓	(55)
2023	Acetylcorynoline	Claudin-1↑, ZO-1↑, Bcl-2↑, Cleaved caspase-3↓, Bax↓, p-AKT↓, p-PI3K↓	(128)
2023	Sakuranetin	TNF-α↓, IL-6↓, IL-17A↓, IL-1β↓, TLR4↓, MyD88↓, p-p65↓, Bcl-2↑, Bax↓, Cleaved caspase-3↓, FITC-dextran↓, I-FABP↓, TEER↑, Occludin↑, ZO-1↑. Significantly attenuated intestinal bacterial translocation to mesenteric lymph nodes, liver, and spleen.	(105)
2023	Vincamine	TNF-α↓, IL-1β↓, IL-6↓, TLR4↓, p-p65↓	(106)
2024	α-cyperone	IL-6↓, TNF-α↓, IL-1β↓, IFN-γ↓, TLR4↓, MyD88↓, p-p65↓, ZO-1↑, Claudin-1↑, FITC-dextran↓, TEER↑. Significantly attenuated intestinal bacterial translocation to mesenteric lymph nodes, liver, and spleen.	(107)
2024	Asperosaponin VI	p-PI3K↓, p-AKT↓, p-p65↓, IL-1β↓, TNF-α↓, ZO-1↑, Claudin-1↑, MUC2↑, Bax↓, Cleaved caspase-3↓, Bcl-2↑	(108)
2024	Sophoricoside	TNF-α↓, IL-1β↓, IL-6↓, IFN-γ↓, FITC-dextran↓, TEER↑, ZO-1↑, Claudin-1↑, Cleaved caspase-3↓, Bax↓, Bcl-2↑, p-PI3K↓, p-AKT↓ Significantly attenuated intestinal bacterial translocation to mesenteric lymph nodes, liver, and spleen.	(129)
2024	Asiaticoside	FITC-Dextran↓, I-FABP↓, TEER↑, TNF-α↓, IL-6↓, IL-1β↓, Ki67↑, Villin↑, Muc2↑, Neurog3↑, SPDEF↑, Significantly attenuated intestinal bacterial translocation to mesenteric lymph nodes, liver, and spleen.	(109)
2024	Isongifolene	TNF-α↓, IFN-γ↓, IL-1β↓, IL-6↓, Cleaved caspase-3/caspase-3↓, Bax↓, Bcl-2↑, p-AMPK↓, p-PGC1α↓, ZO-1↑, Claudin-1↑, TEER↑	(110)
2024	Prunetin	TNF-α↓, IL-6↓, IL-1β↓, TLR4↓, MyD88↓, ZO-1↑, Claudin-1↑	(111)
2024	Shionone	TNF-α↓, IL-6↓, IL-1β↓, BCL-2↑, Bax↓, FITC-dextran↓, I-FABP↓. Significantly attenuated intestinal bacterial translocation to mesenteric lymph nodes, liver, and spleen.	(112)
2024	Bergapten	FITC-dextran↓, I-FABP↓, TEER↑, ZO-1↑, Claudin-1↑, Claudin-2↓, Th17↓, Treg↑, IL-17↓, IFN-γ↓, RORγt↓, T-bet↓, IL-10↑, IL-5↑, Foxp3↑, GATA3↑, Beclin-1↑, Pink1↑, Mito-Parkin↑, LC3II/LC3I↑, p62↓, Cyto-Parkin↓, PPARγ↑, p-p65↓	(56)
2024	Sclareol	FITC-dextran↓, I-FABP↓, MLCK↓, p-MLC/MLC↓, ZO-1↑, Claudin-1↑, IL-1β↓, IL-6↓, TNF-α↓, IFN-γ↓, IL-8↓, p-IκBα↓, p-p65↓, Nrf2↑, HO-1↑. Significantly attenuated intestinal bacterial translocation to mesenteric lymph nodes, liver, and spleen.	(113)
2024	Peiminine	IL-1β↓, IL-6↓, TNF-α↓, IL-17A↓, GSH-Px↑, Bax↓, Cleaved caspase-3↓, Bcl-2↑, ZO-1↑, Claudin-1↑, FITC-dextran↓, TEER↑. Significantly attenuated intestinal bacterial translocation to mesenteric lymph nodes, liver, and spleen.	(114)
2024	Magnolin	TNF-α↓, IL-1β↓, IL-6↓, Bcl-2↑, Bax↓, Cleaved caspase-3↓, p-PI3K↓, p-AKT↓, FITC-dextran↓, IFABP↓, TEER↑, ZO-1↑, Claudin-1↑. Significantly attenuated intestinal bacterial translocation to mesenteric lymph nodes, liver, and spleen.	(115)
2025	Cimifugin	IFN-γ↓, IL-17↓, p-JNK↓, p-ERK↓, p-p38↓, IL-4↑, IL-10↑, Claudin-1↑, ZO-1↑, Th1↓, Th17↓, Th2↑, Treg↑	(57)
2025	Nodakenin	NLRP3↓, GSDMD-N↓, Cleaved caspase-1↓, caspase-11↓, IL-1β↓, IL-18↓, p-PI3K↓, p-Akt↓, FITC-dextran↓, I-FABP↓ Significantly attenuated intestinal bacterial translocation to mesenteric lymph nodes, liver, and spleen.	(130)
2025	Oleic Acid	Bax↓, Bcl-2↑, CD206↑, CD163↑, ZO-1↑, Claudin-1↑, IL-1β↓, IL-6↓, TNF-α↓, GPX4↑, SLC7A11↑, ACSL4↓, GSH↑, SOD↑, MDA↓, ROS↓	(58)
2025	Engeletin	TNF-α↓, IL-1β↓, IL-6↓, Claudin-1↑, Occludin↑, ZO-1↑, iNOS↓, CD86↓, TLR4↓, MyD88↓, TRAF6↓, p-IκBα↓, p-p65↓	(59)
2025	Melezitose	IL-1β↓, IL-12↓, TNF-α↓, IL-10↑, PGK1↓, ATP↓, 2-PG↓,3-PG↓	(116)
2025	Cynaroside	IL-1β↓, TNF-α↓, IL-6↓, Bcl-2↑, Bax↓, Cleaved caspase-3↓, p-PI3K↓, p-AKT↓, p-CREB↓, RXRA↓, ZO-1↑, Claudin-1↑, FITC-dextran↓, TEER↑ Significantly attenuated intestinal bacterial translocation to mesenteric lymph nodes, liver.	(117)

↑, increase; ↓, decrease; ACSL4, acyl-CoA synthetase long-chain family member 4; ATP, adenosine triphosphate; COX-2, cyclooxygenase-2; FITC-dextran, fluorescein isothiocyanate-dextran; G-CSF, granulocyte colony-stimulating factor; GPX4, glutathione peroxidase 4; GSDMD-N, N-terminal fragment of gasdermin D; GSH, glutathione; HO-1, heme oxygenase-1; ICAM-1, intercellular adhesion molecule-1; IEC, intestinal epithelial cell; I-FABP, intestinal fatty-acid-binding protein; IL, interleukin; iNOS, inducible nitric oxide synthase; MCP-1, monocyte chemoattractant protein-1; MDA, malondialdehyde; MLCK, myosin light chain kinase; MPO, myeloperoxidase; MUC2, mucin 2; MyD88, myeloid differentiation primary response 88; NF-κB, nuclear factor-kappa B; NO, nitric oxide; NQO1, NAD(P)H:quinone oxidoreductase 1; PGK1, phosphoglycerate kinase 1; RANTES, regulated on activation, normal T cell expressed and secreted; ROS, reactive oxygen species; SLC7A11, solute carrier family 7 member 11; SOD, superoxide dismutase; TEER, transepithelial electrical resistance; TGF-β, transforming growth factor-beta; TLR4, Toll-like receptor 4; TNF-α, tumor necrosis factor-alpha; TRAF6, TNF receptor-associated factor 6; ZO-1, zonula occludens-1; 2-PG, 2-phosphoglycerate; 3-PG, 3-phosphoglycerate.

## 2 Pathogenesis of Crohn’s disease

### 2.1 Genetic susceptibility

Genome-wide association studies (GWAS) have identified more than 200 susceptibility loci for inflammatory bowel disease (IBD), with the strongest associations converging on pathways involved in bacterial sensing (NOD2), autophagy, and the interleukin-23 (IL-23)/Th17 immune axis (9). In 2001, nucleotide-binding oligomerization domain-containing protein 2 (NOD2) was identified as a CD susceptibility gene, and its mutations impair the recognition of bacterial muramyl dipeptide and autophagic function, leading to weakened responses to bacterial pathogens (10). Autophagy-related genes such as autophagy related 16-like 1 (ATG16L1) and immunity-related GTPase M (IRGM) play critical roles in intracellular pathogen clearance and maintenance of intestinal epithelial cell homeostasis. The CD-associated ATG16L1 Thr300Ala variant disrupts autophagic flux by promoting caspase-3-mediated degradation, thereby impairing intracellular bacterial clearance and Paneth cell function (Paneth cells are specialized intestinal epithelial cells located at the base of the small intestinal crypts that secrete antimicrobial peptides and maintain intestinal barrier homeostasis) (11). IRGM exerts protective effects by regulating autophagy initiation and mediating autophagic degradation of the NOD-like receptor family pyrin domain containing 3 (NLRP3) inflammasome; its deficiency significantly increases susceptibility to chemically induced colitis (12). In addition, leucine-rich repeat kinase 2 (LRRK2) has been identified by GWAS as a major susceptibility gene locus for CD (13). LRRK2 regulates intestinal immune homeostasis through autophagy modulation, and its hyperactivation impairs autophagy and induces Paneth cell dysfunction (14).

### 2.2 Environmental factors

Multiple environmental factors have also been confirmed to influence the risk of CD. Cigarette smoking is the most well-established environmental risk factor, which increases the risk of developing CD and is associated with more severe and refractory disease (15, 16). Antibiotic exposure, particularly early in life, may increase CD risk through disruption of the gut microbiota (17). Among dietary factors, increased consumption of ultra-processed foods is positively associated with the risk of CD, whereas dietary fiber intake exerts a protective effect (18, 19). In addition, breastfeeding and vitamin D are also considered potential protective factors (20). These environmental factors may contribute to CD pathogenesis by altering gut microbiota composition, disrupting the mucosal barrier, or modulating host immune responses.

### 2.3 Intestinal barrier dysfunction

The intestinal epithelial barrier serves as the first line of defense against luminal antigens, microorganisms, and their toxins. Its integrity relies on the tight regulation of paracellular pathways by tight junction proteins (zonula occludens-1 [ZO-1], Occludin, and Claudin), as well as on antimicrobial peptides secreted by Paneth cells and the mucus layer produced by goblet cells (21). In CD, disruption of the intestinal epithelial barrier represents an early and critical event. Gene mutations such as NOD2, ATG16L1, and IRGM can lead to Paneth cell dysfunction by impairing the autophagy pathway, resulting in reduced secretion of antimicrobial peptides including α-defensins (HD-5, HD-6) and lysozyme (22). The deficiency of antimicrobial peptides weakens the mucosal antimicrobial barrier, leading to alterations in gut microbiota composition and bacterial overgrowth at the mucosal surface, which in turn exacerbates inflammatory responses (23). In addition, LRRK2 hyperactivation also indirectly induces Paneth cell dysfunction through autophagy impairment (14). Environmental factors such as cigarette smoking and ultra-processed foods may further compromise the barrier by directly damaging epithelial cells or altering microbiota composition (16).

### 2.4 Immune dysregulation

CD is characterized by a predominantly T helper cell type 1 (Th1)/T helper cell type 17 (Th17)-mediated inflammatory response (24). Th1 responses are driven by interleukin-12 (IL-12) through signal transducer and activator of transcription 4 (STAT4) signaling to induce T-bet, leading to interferon-γ (IFN-γ) secretion. Th17 differentiation is initiated by interleukin-6 (IL-6) and transforming growth factor-β (TGF-β), and is maintained by IL-23 through signal transducer and activator of transcription 3 (STAT3) signaling, resulting in the production of pro-inflammatory cytokines including IL-17A and IL-17F (25). IL-23 is a core factor that sustains the pathogenic function of Th17 cells; upon binding to its receptor, it activates the JAK2/STAT3 pathway, forming a positive feedback loop that amplifies inflammatory responses, damages the intestinal mucosa, and promotes pathological fibrosis (26). The balance between pro-inflammatory Th1/Th17 cells and anti-inflammatory regulatory T cells (Treg) is crucial for maintaining intestinal immune homeostasis. Treg cells exert their suppressive functions through the secretion of IL-10 and TGF-β; their functional impairment or an imbalance in the Th17/Treg ratio is a key driver of CD pathogenesis (27).

### 2.5 Programmed cell death

In recent years, the role of programmed cell death (PCD) in the pathogenesis of CD has garnered increasing attention. Multiple cell death pathways are dysregulated in CD, including apoptosis, necroptosis, pyroptosis, ferroptosis, and autophagy-dependent cell death (28). Apoptosis is mediated through intrinsic (mitochondrial) and extrinsic (death receptor) pathways. TNF-α initiates extrinsic apoptotic signaling upon binding to TNFR, and M1 macrophage infiltration exacerbates intestinal barrier disruption through TNF-α-mediated apoptosis (29). In CD patients, increased intestinal epithelial cell apoptosis is associated with Bcl-2/Bax ratio imbalance and caspase-3 activation (30). Furthermore, circSMAD4 promotes intestinal epithelial cell apoptosis by suppressing Bcl-2 expression via the miR-135a-5p/JAK2 axis (31). Pyroptosis is characterized by gasdermin family protein (GSDMD, GSDME)-mediated plasma membrane pore formation. In CD, NLRP3 inflammasome assembly activates caspase-1, which cleaves GSDMD and releases IL-1β and IL-18; GSDME cleavage by caspase-3 also promotes pyroptosis (32). IRGM exerts protective effects by disrupting NLRP3 and ASC oligomerization and inhibiting inflammasome assembly (33). Circular RNAs play important regulatory roles in pyroptosis: hsa_circRNA_103124 is upregulated in peripheral blood mononuclear cells of CD patients, enhancing NLRP3 and GSDMD expression to promote pyroptosis (34). Ferroptosis is characterized by iron-dependent lipid peroxidation, manifesting as decreased glutathione peroxidase 4 (GPX4) expression and lipid peroxide accumulation in CD (35). Bioinformatics analyses have identified five ferroptosis-related hub genes (PTGS2, IFNG, IL1B, IDO1, and NOS2) that are significantly upregulated in CD (36). Adherent-invasive *Escherichia coli (AIEC)* infection induces ferroptosis in intestinal epithelial cells by reducing GPX4 expression and increasing lipid peroxide accumulation (37). Pro-inflammatory dietary patterns promote ferroptosis susceptibility by downregulating GPX4 expression through endoplasmic reticulum stress-mediated HSPA5 suppression (38). Autophagy-dependent cell death, as a form of type II PCD, also plays an important role in CD pathogenesis. Autophagy is regulated by autophagy-related genes, and its excessive activation can lead to cellular self-digestion and death (39). The CD-associated ATG16L1 Thr300Ala variant disrupts autophagic flux, thereby impairing intracellular bacterial clearance and Paneth cell function, and exacerbating intestinal inflammation (40). IRGM exerts protective effects by regulating autophagy initiation and mediating autophagic degradation of the NLRP3 inflammasome (12). Of note, autophagy serves dual “pro-survival” and “pro-death” functions—physiological autophagy maintains intestinal epithelial homeostasis by clearing damaged organelles and pathogens, whereas excessive activation leads to cell death (28).

### 2.6 Gut microbiota dysbiosis

CD patients exhibit significantly altered gut microbial diversity and composition, characterized by a reduction in beneficial bacteria and expansion of opportunistic pathogens. The butyrate-producing anti-inflammatory bacterium *Faecalibacterium prausnitzii* is consistently depleted in CD patients, even during remission, and its reduction correlates with disease severity (41). Conversely, adherent-invasive *AIEC* is enriched in CD patients; it manipulates host autophagy through mechanisms such as impairing SUMOylation, thereby enhancing its survival within macrophages, and evades host immune clearance through multifaceted virulence factors to promote inflammation (42). Gut dysbiosis disrupts immune homeostasis and triggers the production of pro-inflammatory cytokines such as IL-17 and IL-23, further exacerbating the inflammatory process (43).

The interplay among genetic, environmental, barrier, immune, cell death, and microbial factors collectively drives the initiation and progression of CD. A comprehensive understanding of these pathogenic mechanisms provides the necessary pathophysiological context for understanding how TCM exerts multi-target interventions in CD. The following sections will systematically elaborate on the mechanistic evidence for TCM in treating CD through multiple pathways, including regulating immune balance, suppressing inflammatory responses, protecting intestinal epithelial cells, reinforcing the mucosal barrier, and restoring microbial homeostasis.

## 3 Mechanisms of TCM in treating Crohn’s disease

### 3.1 Regulation of immune balance

Immune dysregulation plays a pivotal role in the pathogenesis of Crohn’s disease. Notably, helper T cells are essential for maintaining intestinal immune homeostasis. Differentiation of Th1 cells is primarily driven by IL-12 and IFN-γ, which collectively promote the conversion of naïve T cells into Th1 cells and trigger inflammatory responses that exacerbate intestinal inflammation (44). Th17 cells play a critical role in autoimmune responses. Specifically, they secrete multiple pro-inflammatory mediators, including TNF-α, IL-17, and IL-6, that promote mucosal inflammation (45). Meanwhile, regulatory T (Treg) cells secrete IL-10 and TGF-β to inhibit the proliferation of other Th subsets and suppress responses to self-antigens, thereby mitigating environmentally induced mucosal inflammation. Th17 and Treg cells can also interconvert under specific conditions (46). A balanced equilibrium between Th1/Th17 and Treg cells is crucial for governing intestinal immune responses (47).

The Jianpi formula down-regulates IFN-γ to restore Th1/Th2 balance, as well as decreases IL-17 and RORγt while increasing FoxP3 to restore Th17/Treg balance, thereby preventing TNBS-induced CD in rats (48). Triptolide attenuates Th17-mediated immunity by regulating the IL-6/STAT3 pathway and minimizing lamina propria T-cell infiltration, thereby alleviating intestinal inflammation (49). Artesunate suppresses Th1/Th17-mediated inflammation through the NF-κB pathway and mitigates the experimental CD in models (50). Tripterygium wilfordii glycoside increases Treg abundance in diseased intestinal segments, modulates the local immune microenvironment, and alleviates intestinal inflammation (51). Berberine restores the M1/M2 macrophage ratio and rebalances Th1/Th17/Treg subsets, thereby ameliorating TNBS-induced colitis (52). Clematichinenoside AR modulates the Treg/Th17 balance via the PI3K/Akt pathway and attenuates CD symptoms (53). Eriocalyxin B attenuates M1 macrophage polarization by inhibiting the JAK2/STAT1 pathway and improves intestinal inflammation (54). Diosmetin potentially modulates the Th1/Th2 and Th17/Treg balance through PI3K/AKT inhibition, ameliorating mucosal immune dysregulation and the experimental CD in models (55). Bergapten reduces the Th17/Treg ratio by decreasing the proportion of Th17 cells and increasing that of Tregs, thereby improving intestinal inflammation (56). Earlier studies have established that cimifugin may modulate Th1/Th2 and Th17/Treg balance in murine intestines by suppressing MAPK activation (57). Oleic acid promotes M2 macrophage polarization, attenuates inflammation, and improves CD pathology (58). Lastly, engeletin suppresses M1 macrophage polarization via TLR4/NF-κB inhibition and attenuates inflammatory responses (59). Collectively, these findings demonstrate that TCM exerts profound immunomodulatory effects by simultaneously targeting multiple axes of the dysregulated immune network in CD, thereby restoring homeostasis and curtailing chronic inflammation.

### 3.2 Inhibition of inflammatory responses

CD is a chronic inflammatory bowel disorder with a complex pathogenesis, wherein dysregulated expression of inflammatory cytokines represents a central pathogenic factor (60). In CD patients, excessive secretion of pro-inflammatory cytokines such as TNF-α, IL-1β, IL-6, IL-12, and IL-23 activates immune cells, perpetuates a vicious cycle of inflammation, and exacerbates mucosal injury, ultimately culminating in chronic intestinal inflammation (61). TNF-α is a key pro-inflammatory mediator that facilitates inflammatory cell infiltration and cytokine release. In contrast, IL-1β and IL-6 initiate early inflammatory responses and amplify inflammatory signaling, whereas IL-12 and IL-23 perpetuate inflammation by directing T-cell subset differentiation (62). Aberrant expression of these cytokines not only underlies chronic mucosal inflammation but also predisposes to complications such as fistulas and strictures (63).

Daikenchuto up-regulates endogenous adrenomedullin in the intestine, suppresses elevated serum amyloid A levels in plasma, and attenuates increases in the levels of colonic pro-inflammatory cytokines TNF-α and IFN-γ, thereby mitigating mucosal damage and inflammatory adhesions. Moreover, it alleviates visceral pain via TRP channels and selectively limits eosinophil infiltration (64, 65). Baizhu Fuling Decoction decreases TNF-α, IL-1β, IL-6, and IL-8 while restoring IL-4 and IL-10 to normal levels in CD rats, likely via the TLR4/NF-κB pathway (66–70). At the same time, Xianglian Wan ameliorates symptoms in CD model rats, an effect associated with reduced TNF-α and IL-1β levels and a concomitant increase in the level of the anti-inflammatory cytokine IL-4 (67). Both Huangqijiegeng Decoction and Huangqihuanglian Decoction downregulate colonic ICAM-1 expression and attenuate local immune injury in CD rats (71). An herbal extract derived from Abelmoschus manihot and Prunus mume has demonstrated therapeutic efficacy in the treatment of CD, and its mechanism potentially involves reducing colonic TNF-α and IFN-γ levels (72). The Jian-Pi formula attenuates TNBS-induced colonic inflammation in CD rats, a process linked to suppressed expression of NF-κB p65, IL-23, and CCL20/CCR6 (73). Herbal enemas have been extensively employed in the treatment of CD. For example, Kuijiekang lowers IFN-γ and IL-1 levels while increasing IL-4 and IL-10 levels, thus suppressing inflammation and alleviating CD (74). Shenling Baizhu Powder reduces IL-1β, IL-1, and IL-17C levels and increases IL-4 and IL-10 levels, thereby attenuating inflammation and promoting mucosal repair (75, 76). Additional studies have revealed that Shenling Baizhu Powder down-regulates the p38 MAPK pathway, decreases TNF-α, IL-1, and IL-6 levels, and mitigates intestinal inflammation in CD rats (77). Concurrently, Shenling Baizhu Powder alleviates intestinal inflammation in CD rats, an effect potentially mediated by PI3K/Akt pathway inhibition (78). Tou Nong San decreases the levels of TNF-α, IL-6, IL-1β, and IL-17 by targeting NF-κB, thereby attenuating inflammation in CD rat models (79). Xue-Jie-San may inhibit intestinal autophagy by mediating the mTOR/Beclin1 pathway, thereby limiting inflammasome activation and pro-inflammatory cytokine expression and ameliorating colitis in CD rats (80). Xue-Jie-San may suppress Notch1 signaling, enhance Nrf2/GPX4 activity, decrease lipid peroxidation products (e.g., 4-HNE), and reduce pro-the levels of inflammatory cytokines (IL-6, IL-17, TNF-α), thereby ameliorating experimental CD in rats (81). Moreover, Xue-Jie-San is a potential agent for CD complicated by atherosclerosis, possibly through inhibiting the TLR4/MyD88/NF-κB pathway and suppressing pro-inflammatory cytokines, chemokines, and adhesion molecules (82). Huanghou Zhixie Dropping Pills suppress cytokine release via PI3K/AKT/NF-κB modulation and inhibit activated T cells via PI3K/Akt suppression, thereby attenuating inflammation and improving CD symptoms in rats (83). Qinghua Xiaoyong Formula attenuates PI3K/AKT signaling, decreases TNF-α, IL-6, and IL-1β levels, and ameliorates CD-like symptoms in mice (84). Changyanning tablets alleviate CD by downregulating TNF-α and IL-6 levels (85). Xiangsha Liujunzi Wan attenuates PI3K-AKT and MAPK signaling, reduces TNF-α, IL-6, and IL-1β levels, suppresses inflammation, and mitigates TNBS-induced colitis (86).

Furthermore, pomegranate extract inhibits the MAPK and NF-κB signaling pathways, decreases the levels of downstream inflammatory mediators, and alleviates TNBS-induced colitis (87). Rosa laevigata fruit extract lowers colonic TNF-α and IFN-γ levels and protects the intestinal mucosa in CD mice (88). Brucea javanica oil suppresses TLR4/NF-κB activation, reduces the levels of pro-inflammatory mediators (TNF-α, IL-1β, IL-6, MPO), increases those of anti-inflammatory cytokines (IL-4, IL-10, TGF-β), and downregulates MMP-1, MMP-3, and RAGE expression, thereby protecting colonic structural integrity (89). Mume Fructus attenuates inflammation by reducing IL-1β, IL-6, IL-17, MPO, TGF-β, and LPS levels, thereby alleviating intestinal inflammation in CD rats (90). Both Mume powder and its aqueous decoction suppress the expression of NO, IL-1β, IL-6, and IL-17 while increasing that of IL-10, thereby improving CD symptoms in rats (91).

Triptolide inhibits the TLR/NF-κB pathway in both colonic tissue and cultured cells from CD patients, suppresses Th1-type cytokines, attenuates inflammation, and exerts therapeutic effects in CD (92). Ellagic acid has been reported to attenuate the MAPK and NF-κB signaling pathways, suppress the expression of inflammatory mediators (MPO, COX-2, iNOS), and minimize tissue damage in CD rat models (93). Artesunate inhibits NF-κB activation, decreases pro-inflammatory cytokines (TNF-α, IL-17, IFN-γ), increases the anti-inflammatory cytokine IL-10, and markedly ameliorates TNBS-induced colitis (50). Tripterygium glycosides attenuate inflammatory responses by inhibiting the TGF-β/Smad pathway and exert therapeutic effects in CD rats (94). Berberine inhibits NF-κB activation, decreases the levels of pro-inflammatory cytokines (IFN-γ, IL-17, IL-6, IL-1β, TNF-α), and increases the level of the anti-inflammatory cytokine IL-10, thereby mitigating TNBS-induced colitis (52). Baicalein directly binds myeloid differentiation protein-2 (MD-2), disrupts the TLR4/MyD88 complex, inhibits the NF-κB and MAPK signaling pathways, and markedly down-regulates the expression of pro-inflammatory cytokines (95). Quercitrin decreases TNF-α and IL-1β levels and ameliorates intestinal injury in CD rats (96). Total flavone of Abelmoschus manihot suppresses the NF-κB and MAPK pathways, reduce the levels of TNF-α, IFN-γ, IL-6, IL-1β, IL-12, IL-17 and MPO, and attenuates TNBS-induced colitis in mice (97, 98). Procyanidin B2 has been documented to exert anti-inflammatory effects by inhibiting the intestinal PI3K/AKT pathway, decreasing TNF-α and IL-1β release, and increasing IL-10 production (99). Clematichinenoside AR downregulates the expression of IL-1β, TNF-α, and IL-17A via the PI3K/Akt pathway and exerts therapeutic effects in CD (53). Tetramethylpyrazine attenuates TNBS-induced CD in rats by suppressing the expression of IL-2, IL-12, IFN-γ, TNF-α, ICAM-1, iNOS, and P-selectin (100). Loureirin B inhibits the expression of IL-1, IL-1β, IL-6, TNF-α, TGF-β, and MPO via IL-6/STAT3/NF-κB modulation, up-regulates IL-10 expression, and exerts anti-inflammatory effects (101). Ruscogenins activate the Nrf2/NQO1 pathway, decrease TNF-α and IFN-γ levels, inhibit macrophage-migration-related factors, and ameliorate murine colitis (102). Pachymic acid inhibits the PI3K/AKT signaling pathway, reduces TNF-α, IL-6, and IL-1β levels, and alleviates CD in mice (103). Phillygenin suppresses the IL-17 signaling pathway and subsequent NF-κB activation, decreases TNF-α, IL-6, and IFN-γ levels, and improves experimental CD in mouse models (104). Eriocalyxin B lowers IL-6, TNF-α, and IL-1β levels by inhibiting the JAK2/STAT1 pathway and ameliorates intestinal inflammation (54). According to an earlier study, diosmetin attenuates experimental CD in murine models by inhibiting PI3K/AKT signaling and reducing the levels of TNF-α, IFN-γ, and IL-17A (55). Sakuranetin has been shown to attenuate intestinal inflammation by suppressing TLR4/NF-κB activation and decreasing the levels of TNF-α, IL-6, IL-17A, and IL-1β (105). Vincamine modulates the TLR4/NF-κB signaling pathway, reduces TNF-α, IL-1β, and IL-6 levels, and ameliorates TNBS-induced colitis in mice (106). α-Cyperone modulates TLR4/NF-κB signaling to down-regulate the expression of IL-6, TNF-α, IL-1β and IFN-γ, alleviating experimental CD in models (107). Asperosaponin VI suppresses the PI3K/AKT/NF-κB pathway, significantly reducing colonic IL-1β and TNF-α levels in TNBS-induced mouse models (108). Sophoricoside inhibits the PI3K/AKT signaling pathway, lowers TNF-α, IL-1β, IL-6 and IFN-γ levels, and suppresses inflammatory responses (109). Asiaticoside decreases TNF-α, IL-6 and IL-1β levels, thereby attenuating experimental CD in mouse models (109). Isongifolene reduces TNF-α, IFN-γ, IL-1β and IL-6 levels, alleviating colitis symptoms in experimental CD in mouse models (110). Prunetin inhibits the TLR4/MyD88 pathway, reduces TNF-α, IL-6 and IL-1β levels, mitigates intestinal inflammation, and improves experimental CD in mouse models (111). Shionone decreases TNF-α, IL-6 and IL-1β levels, thereby alleviating experimental CD in mouse models (112). Sclareol modulates Nrf2/NF-κB/MLCK signaling, reduces IL-1β, IL-6, TNF-α, IFN-γ, and IL-8 levels, and suppresses inflammation (113). Peiminine activates the Nrf2/HO-1 pathway, inhibits IL-1β, IL-6, TNF-α and IL-17A levels, and attenuates inflammatory responses (114). Magnolin inhibits PI3K/AKT signaling, suppresses TNF-α, IL-1β and IL-6 levels, and alleviates intestinal inflammation (115). Cimifugin suppresses MAPK activation, modulates immune balance, and concomitantly decreases IFN-γ and IL-17 levels, increases IL-4 and IL-10 levels, thereby inhibiting inflammation (57). Oleic acid inhibits IL-1β, IL-6 and TNF-α levels, alleviating intestinal inflammation (58). Engeletin suppresses the TLR4-NF-κB pathway, reduces TNF-α, IL-1β and IL-6 levels, and alleviates intestinal inflammation (59). Melezitose inhibits the activity of the glycolytic enzyme PGK1, decreases IL-1β, IL-12 and TNF-α levels, and increases IL-10 levels, thereby alleviating inflammatory responses (116). Finally, cynaroside inhibits PI3K/AKT activation, suppresses TNF-α, IL-6 and IL-1β levels, and exerts anti-inflammatory effects (117). The broad suppression of key inflammatory signaling hubs and cytokine cascades by diverse TCM agents highlights a fundamental capacity to disrupt the self-perpetuating cycle of inflammation that defines CD pathogenesis.

### 3.3 Protection of the intestinal barrier

The intestinal mucosal barrier constitutes the first line of defense against luminal antigens, microbes, and their toxins, and comprises four inter-related components (118). The physical barrier is formed by a monolayer of intestinal epithelial cells and their apical tight-junction proteins, encompassing claudin, Occludin, and zonula occludens-1 (ZO-1), creating a selectively permeable barrier that prevents the entry of pathogens and harmful substances while permitting nutrient absorption (119). The chemical barrier is composed of secreted factors such as gastric acid, bile, digestive enzymes, antimicrobial peptides produced by Paneth cells, and MUC2 mucin secreted by goblet cells, which together establish a chemical defense against pathogens (120, 121). The immune barrier encompasses both innate and adaptive immune systems, which achieve precise pathogen clearance and controlled inflammatory responses through antibody secretion, phagocytosis, and balanced immune-cell regulation (122). The microbial barrier is composed of commensal gut microbiota, which maintains micro-ecological balance and promotes epithelial repair by competing for nutrients, secreting antimicrobial substances, and generating metabolites such as short-chain fatty acids (123, 124). In summary, an intact intestinal mucosal barrier is an essential protective and defensive system for the organism. Disruption of this barrier underlies the development of inflammatory bowel disease, alters the intestinal microenvironment, skews immune responses, and exacerbates inflammation, thereby accelerating CD progression (125). Therefore, maintaining and restoring intestinal barrier integrity represents a crucial therapeutic strategy in CD management.

Shenling Baizhu Powder may improve intestinal barrier function in CD rats by modulating miR-21, miR-150, and miR-200b expression, thereby regulating Claudin-1 protein levels (126). It also enhances colonic barrier repair in CD rats by up-regulating the mRNA and protein expression of Occludin, ZO-1, and E-cadherin (127). Changyanning tablets protect the intestinal mucosal barrier by regulating Occludin and Claudin-1 expression and alleviate CD (85). Xiangsha Liujunzi Wan suppresses the PI3K-AKT and MAPK signaling pathway, increases ZO-1 and Occludin levels, protects barrier integrity, and mitigates TNBS-induced colitis (86). Mume Fructus not only increases ZO-1 and Occludin levels but also improves microbial richness and restores short-chain fatty acid (SCFA) production, thereby preserving the integrity of the intestinal mucosal barrier (90). Procyanidin B2 has been hypothesized to protect murine mucosal barrier function by decreasing FITC-dextran levels and limiting bacterial translocation to mesenteric lymph nodes (MLNs), while simultaneously upregulating Claudin-1 and ZO-1 expression to reinforce barrier structure (99). Clematichinenoside AR prevents bacterial translocation to the liver and MLNs, decreases intestinal permeability, and up-regulates Occludin and ZO-1 expression to protect the mucosal barrier (53). Ruscogenins decrease FITC-dextran levels, increase transepithelial electrical resistance (TEER), and inhibit bacterial translocation to the liver and MLNs, thereby ameliorating TNBS-induced barrier dysfunction (102). Pachymic acid decreases serum FITC-dextran and intestinal fatty-acid-binding protein (I-FABP) levels, increases TEER, and preserves the intestinal mucosal barrier in mice (103). Acetylcorynoline increases Claudin-1 and ZO-1 levels in both colonic tissue and colonoid cultures, thereby protecting barrier architecture (128). Sakuranetin reduces FITC-dextran and I-FABP levels, increases TEER, and suppresses bacterial translocation to the liver, spleen, and MLNs, thereby safeguarding the intestinal mucosal barrier (105). Similarly, α-Cyperone decreases intestinal FITC-dextran permeability, increases TEER, reduces bacterial translocation to the liver, spleen, and MLNs, and up-regulates Claudin-1 and ZO-1 to protect the mucosal barrier (107). Asperosaponin VI increases the expression levels of ZO-1, Claudin-1, and the mucus protein MUC2, thereby preserving intestinal barrier integrity (108). Sophoricoside decreases FITC-dextran levels, increases TEER, up-regulates the expression of ZO-1 and Claudin-1, and reduces bacterial translocation to the liver, spleen, and MLNs (129). Asiaticoside enhances intestinal stem-cell proliferation and differentiation, inhibits bacterial translocation to the liver, spleen, and mesenteric lymph nodes, lowers FITC-dextran and I-FABP leakage, and increases TEER, thereby strengthening the epithelial barrier (109). Isongifolene increases the expression levels of ZO-1, Claudin-1, and TEER, thereby improving barrier function in TNBS-treated mice (110). Prunetin suppresses the TLR4/MyD88 pathway to attenuate inflammation, as well as up-regulates the expression of ZO-1 and Claudin-1, thereby protecting the intestinal mucosal barrier (111). Shionone decreases FITC-dextran and I-FABP levels, limits bacterial translocation to the liver, spleen, and MLNs, and protects the mucosal barrier (112). Bergapten reduces FITC-dextran and I-FABP levels, increases TEER, decreases mucosal permeability, up-regulates ZO-1 and Claudin-1 expression, and down-regulates claudin-2 expression, thereby protecting the intestinal barrier (56). Sclareol modulates the Nrf2/NF-κB/MLCK signaling pathway, decreases FITC-dextran and I-FABP levels, mitigates bacterial translocation to MLNs, liver, and spleen, and increases the expression levels of ZO-1 and Claudin-1 to preserve barrier integrity (113). Likewise, peiminine decreases FITC-dextran levels, increases TEER, reduces bacterial translocation to MLNs, spleen, and liver, and up-regulates ZO-1 and Claudin-1 via the Nrf2/HO-1 pathway (114). In addition, magnolin decreases FITC-dextran and I-FABP levels, increases TEER, suppresses bacterial translocation, lowers mucosal permeability, and up-regulates ZO-1 and Claudin-1 expression to protect the mucosal barrier (115). Cimifugin has been described to ameliorate barrier injury by suppressing MAPK activation, modulating immunity, inhibiting inflammatory responses, and increasing Claudin-1 and ZO-1 expression (57). Nodakenin decreases FITC-dextran and I-FABP levels, prevents bacterial translocation to MLNs, spleen and liver, and lowers mucosal permeability (130). Engeletin increases Claudin-1, Occludin, and ZO-1 expression and attenuates barrier damage (59). Cynaroside up-regulates the expression of ZO-1 and Claudin-1, decreases serum FITC-dextran levels, increases intestinal TEER, and limits bacterial translocation and mucosal permeability (117). Ultimately, the reinforcement of the intestinal barrier by TCM represents a cornerstone of its therapeutic action, as it directly addresses the fundamental defect of “leaky gut,” preventing the influx of luminal triggers and breaking the cycle of inflammation (**Figure 1**).

**Figure 1:**
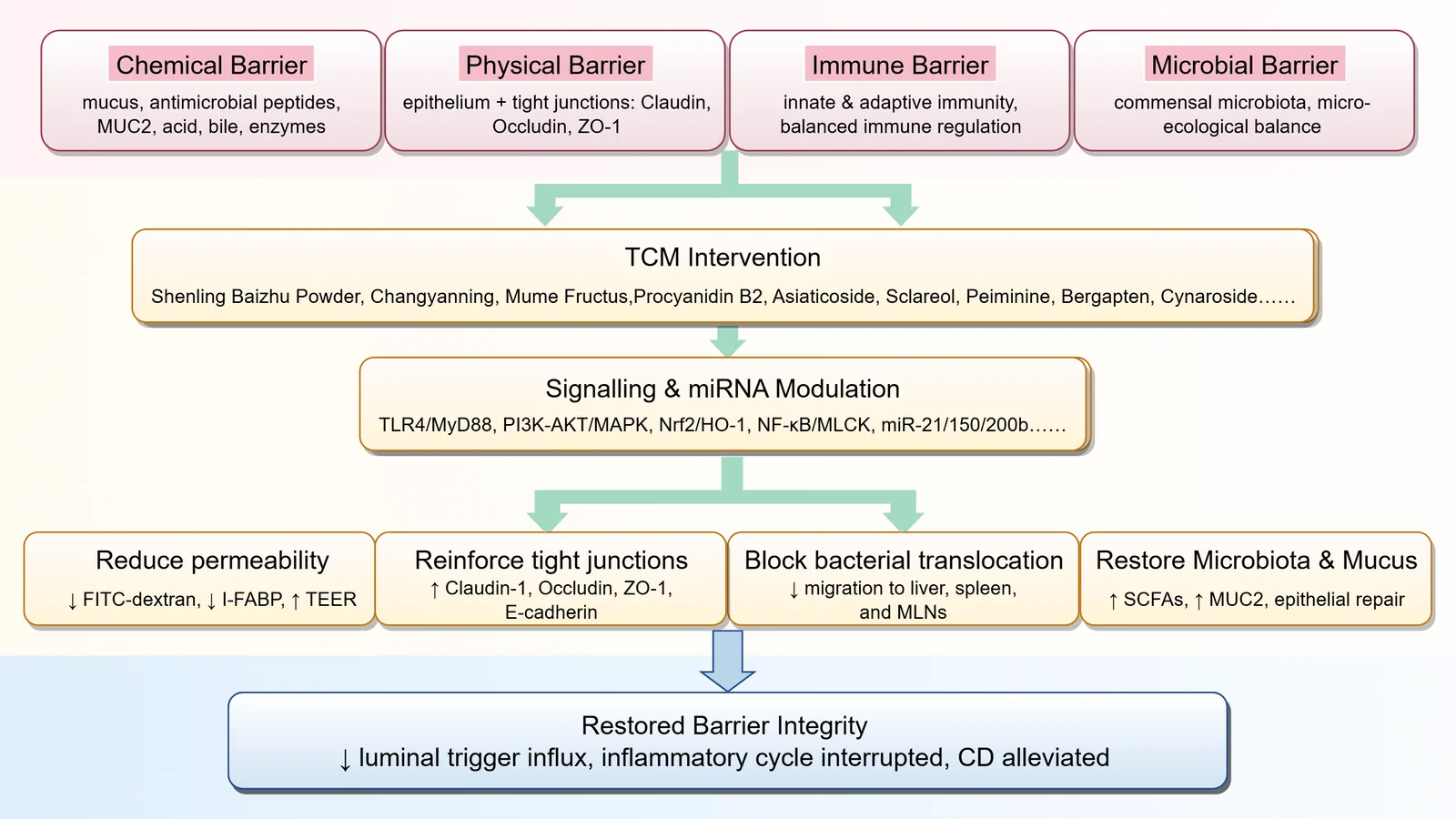
Protection of the intestinal barrier. The right panel illustrates the four components of the intestinal mucosal barrier (chemical, physical, immune, and microbial barriers). The left panel shows TCM formulas and active compounds that exert barrier-protective effects through modulation of signaling pathways (TLR4/MyD88, PI3K-AKT/MAPK, Nrf2/HO-1, NF-κB/MLCK) and miRNAs (miR-21/150/200b). These actions reinforce tight junctions (↑ Claudin-1, Occludin, ZO-1, E-cadherin), reduce permeability (↓ FITC-dextran, ↓ I-FABP, ↑ TEER), block bacterial translocation (↓ migration to liver, spleen, and MLNs), and restore microbiota and mucus (↑ SCFAs, ↑ MUC2, epithelial repair), ultimately restoring barrier integrity, breaking the inflammatory cycle, and alleviating CD.

### 3.4 Regulation of cell death pathways

Abnormal death of intestinal epithelial cells is a core link in disrupting the mucosal barrier and driving chronic inflammation in Crohn’s disease. In addition to modulating immune and inflammatory responses, traditional Chinese medicine also exhibits significant regulatory effects on various programmed cell death pathways. This section will elaborate on the specific mechanisms and evidence of how TCM components inhibit apoptosis, pyroptosis, ferroptosis, and regulate autophagy homeostasis (**Figure 2**).

**Figure 2:**
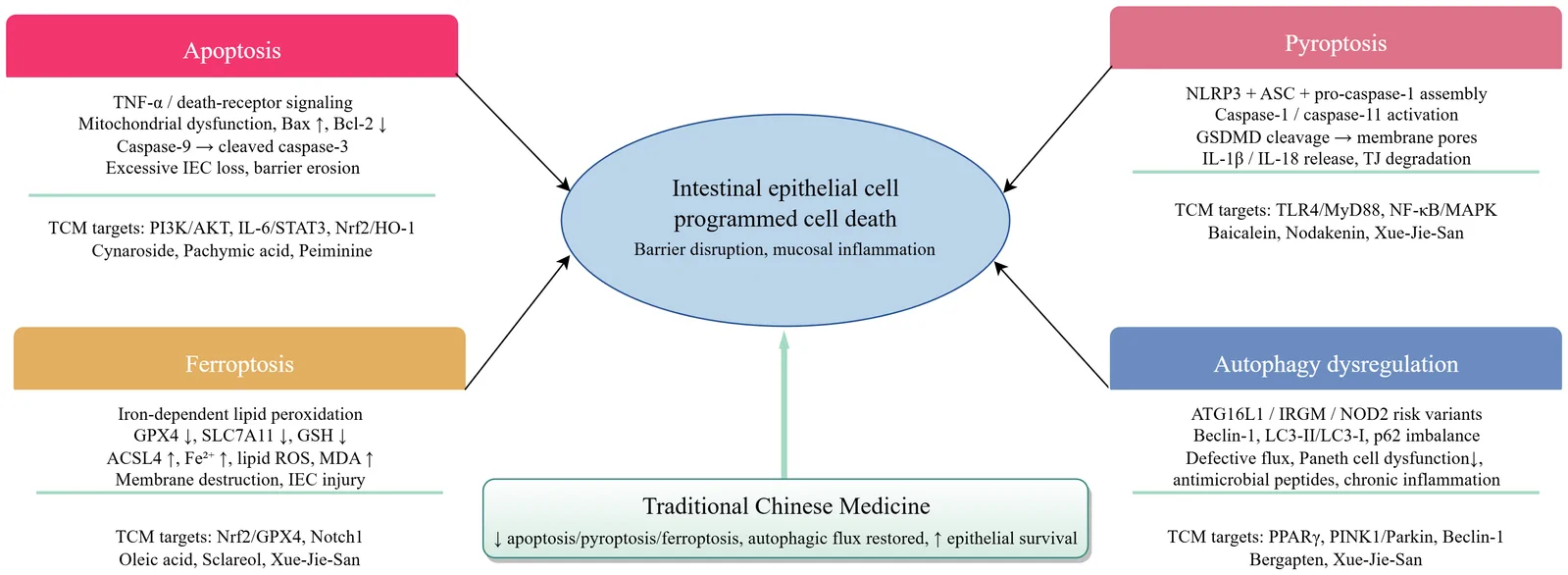
Regulation of cell death pathways. CD involves dysregulation of four major cell death pathways. Apoptosis is mediated by TNF-α/death-receptor signaling, mitochondrial dysfunction, Bax/Bcl-2 imbalance, and caspase-9/caspase-3 activation, leading to excessive IEC loss and barrier erosion; pyroptosis is driven by NLRP3 inflammasome assembly, caspase-1/11 activation, GSDMD cleavage and pore formation, and IL-1β/IL-18 release; ferroptosis is characterized by iron-dependent lipid peroxidation, GPX4/SLC7A11 downregulation, GSH depletion, ACSL4 upregulation, and ROS/MDA accumulation; autophagy dysregulation involves ATG16L1/IRGM/NOD2 risk variants, Beclin-1/LC3-II/p62 imbalance, defective autophagic flux, and Paneth cell dysfunction. TCM formulations and active compounds exert protective effects by modulating these pathways—Cynaroside, Pachymic acid, Peiminine, and Magnolin inhibit apoptosis; Baicalein, Nodakenin, and Xue-Jie-San suppress pyroptosis; Oleic acid, Sclareol, and Xue-Jie-San inhibit ferroptosis; Bergapten, Xue-Jie-San, and Total flavone of Abelmoschus manihot regulate autophagy. These actions collectively reduce intestinal epithelial cell death, preserve barrier integrity, and attenuate mucosal inflammation.

#### 3.4.1 Anti-apoptotic mechanisms

Of note, apoptosis plays a pivotal role in the pathogenesis of Crohn’s disease (131). Previous studies have demonstrated that excessive apoptosis of intestinal epithelial cells (IECs) is a major contributor to the disruption of intestinal barrier structure and function. In CD patients, overexpression of inflammatory cytokines (e.g., TNF-α, IL-6) promotes IEC apoptosis, further increasing intestinal permeability and driving the translocation of harmful substances and bacteria into the intestinal wall and circulation, thereby triggering and sustaining intestinal inflammation (132). Moreover, up-regulation of pro-apoptotic proteins (e.g., Bax, cleaved caspase-3) and the simultaneous down-regulation of anti-apoptotic proteins (e.g., Bcl-2) further validate the central role of apoptosis in CD pathogenesis. Therefore, inhibiting excessive IEC apoptosis is regarded as a vital strategy to preserve intestinal barrier integrity and alleviate intestinal inflammation.

Clematichinenoside AR suppresses the PI3K/Akt pathway, increases Bcl-2 levels, downregulates caspase-3 and Bax expression, inhibits IEC apoptosis, restores intestinal barrier function, and alleviates experimental CD in mouse models (53). Tetramethylpyrazine protects the intestinal mucosa by inhibiting apoptosis of lamina propria mononuclear cells (100). Loureirin B attenuates IL-6/STAT3/NF-κB signaling, up-regulates Bcl-2 expression, down-regulates the expression of Bax, cleaved caspase-3, and cleaved caspase-9, suppresses colonic epithelial apoptosis, ameliorates mucosal damage, and alleviates experimental CD in models (101). Ruscogenin activates the Nrf2/NQO1 pathway, up-regulates Bcl-2 expression, down-regulates the expression of Bax and cleaved caspase-3, inhibits IEC apoptosis, restores barrier function, and alleviates experimental CD in mouse models (102). Pachymic acid inhibits the PI3K/AKT pathway and reduces IEC apoptosis (103). Acetylcorynoline inhibits the PI3K/AKT signaling pathway, decreases the expression of pro-apoptotic proteins (cleaved caspase-3, Bax), and increases the expression of anti-apoptotic protein (Bcl-2), thereby suppressing IEC apoptosis (128). Importantly, a prior investigation has indicated that ssakuranetin potentially inhibits IEC apoptosis by suppressing TLR4/NF-κB activation, up-regulating Bcl-2 expression, and down-regulating the expression of Bax and cleaved caspase-3, thereby attenuating intestinal barrier dysfunction in experimental CD in mouse models (105). Asperosaponin VI suppresses the PI3K/AKT/NF-κB signaling pathway, up-regulates Bcl-2 expression, down-regulates the expression of Bax and cleaved caspase-3, inhibits IEC apoptosis, and protects intestinal barrier integrity (108). Sophoricoside inhibits the PI3K/AKT signaling pathway, up-regulates Bcl-2 expression, down-regulates the expression of Bax and cleaved caspase-3, minimizes IEC apoptosis, improves barrier function, and alleviates experimental CD in animal models (129). Isongifolene activates the AMPK/PGC1-α pathway, down-regulates the expression of pro-apoptotic proteins (cleaved caspase-3, Bax), up-regulates the expression of anti-apoptotic protein Bcl-2, decreases IEC apoptosis, and improves barrier function (110). Shionone down-regulates the expression of Bax, up-regulates that of Bcl-2, and inhibits IEC apoptosis, thereby improving intestinal barrier function (112). Peiminine directly inhibits IEC apoptosis by activating the Nrf2/HO-1 pathway and modulating the expression of apoptosis-related proteins (Bcl-2, Bax, caspase-3) (114). Magnolin inhibits the PI3K/AKT signaling pathway, modulates Bcl-2/Bax/caspase-3 expression, directly limits IEC apoptosis, and protects the mucosal barrier (115). Oleic acid modulates the Bcl-2/Bax balance by up-regulating the expression of Bcl-2 and concomitantly down-regulating that of Bax, thereby inhibiting IEC apoptosis and improving barrier function (58). Cynaroside inhibits PI3K/AKT activation, modulates the Bcl-2/Bax balance and caspase-3 activity, suppresses IEC apoptosis, preserves intestinal barrier integrity, and alleviates inflammatory injury in an experimental CD model (117). Moreover, aberrant survival of immune cells contributes to chronic intestinal inflammation. Triptolide mitigates CD-induced inflammation by suppressing the IL-6/STAT3 signaling pathway, down-regulating the expression of anti-apoptotic factors (Bcl-2, Bcl-xl), and restoring T-cell apoptotic capacity (49). Therefore, the anti-apoptotic properties of TCM constituents serve a dual protective role: preserving the vital intestinal epithelial barrier and reinstating immune tolerance, both of which are crucial for disease control.

#### 3.4.2 Anti-pyroptotic mechanisms

Recently, pyroptosis, an inflammatory programmed cell death mediated by Gasdermin D (GSDMD), has been identified as being markedly activated in CD lesions. In intestinal biopsies from CD patients, the expression level of the pyroptosis-executing N-terminal fragment of GSDMD is elevated and positively correlated with endoscopic and clinical disease activity indices (133). Upon sensing dysbiosis or oxidative stress, the NLRP3 inflammasome assembles and recruits ASC and pro-caspase-1. Activated caspase-1 cleaves GSDMD, generating an N-terminal pore-forming domain that creates membrane pores, leading to cellular swelling, rupture, and massive release of pro-inflammatory cytokines (e.g., IL-1β and IL-18) (134, 135). Pyroptosis not only induces direct loss of IECs and degradation of tight-junction proteins, increasing intestinal permeability, but also promotes translocation of bacteria and their products into the lamina propria, triggering systemic inflammation (136).

Xue-Jie-San suppresses mTOR/Beclin1-mediated autophagy and NLRP3/AIM2 inflammasome activation, down-regulates the expression of the caspase-1/IL-1β/IL-18 axis, effectively inhibits pyroptosis, and ameliorates colonic inflammation in CD (80). Baicalein directly binds MD-2, disrupts the TLR4/MyD88 complex, inhibits the NF-κB and MAPK signaling pathways, and suppresses NLRP3 inflammasome activation and the downstream caspase-1/IL-1β axis, thereby effectively inhibiting pyroptosis and attenuating TNBS-induced colitis (95). Nodakenin inhibits the PI3K/Akt signaling pathway, NLRP3 inflammasome activation, GSDMD-N-mediated pore formation, and caspase-1/11-dependent IL-1β/IL-18 release, thereby effectively inhibiting IEC pyroptosis and improving TNBS-induced colitis and barrier dysfunction (130). Targeting pyroptosis thus emerges as a potent strategy by which TCM can achieve dual benefits: reducing inflammatory cell death and mitigating the release of potent pro-inflammatory cytokines, thereby alleviating tissue damage.

#### 3.4.3 Inhibition of ferroptosis

Recently, ferroptosis, a novel iron-dependent, programmed cell death driven by lipid-peroxide accumulation, has been reported to contribute to IEC barrier disruption and immune-cell dysfunction in CD (137). In the mucosal tissues of patients, glutathione peroxidase 4 (GPX4) and GSH levels are markedly reduced, whereas lipid ROS and transferrin-receptor expression are elevated, sensitizing IECs and macrophages to oxidative stress (35). Bioinformatic analyses further uncovered that ferroptosis-related genes such as PTGS2 and CD44 are positively correlated with M1 macrophage infiltration (138). In animal models, ferroptosis inhibitors attenuate colitis pathology by restoring GPX4 activity (139). Overall, these findings conjointly suggest that targeting ferroptotic pathways may offer novel precision diagnostic and therapeutic strategies for CD.

Oleic acid inhibits ferroptosis by up-regulating the expression of GPX4 and SLC7A11 and down-regulating that of ACSL4, thereby alleviating oxidative stress and intestinal inflammation in CD (58). Xue-Jie-San restores Nrf2/GPX4 axis function by targeting Notch1, inhibiting ferroptosis, and alleviating CD-related inflammation (81). Changyanning tablets up-regulate the expression of GPX4, restore SLC7A11-mediated cystine uptake, and interrupt the lipid ROS–Fe^2+^ positive-feedback loop triggered by GPX4 inactivation, thereby inhibiting IEC ferroptotic cell death (85). The emerging role of TCM in inhibiting ferroptosis opens a novel therapeutic avenue for protecting intestinal epithelial cells from this unique, iron-driven form of destruction, further expanding its repertoire in regulating cell fate in CD.

#### 3.4.4 Regulation of autophagy

In inflammatory bowel disease, autophagy has been extensively shown to modulate disease initiation and progression through immune and inflammatory regulation. Polymorphisms in the autophagy-related genes ATG16L1, IRGM, and NOD2 are significantly associated with CD susceptibility (140). Noteworthily, autophagy plays a crucial role in CD pathogenesis by maintaining IEC homeostasis, regulating antimicrobial-peptide secretion, and eliminating intracellular pathogens (141). Bergapten activates PPARγ, suppresses NF-κB phosphorylation, up-regulates the expression of PINK1, Parkin, Beclin-1, and LC3-II/LC3-I, down-regulates that of p62, promotes mitophagy, and ameliorates CD in experimental mouse models (56). Xue-Jie-San potentially suppresses intestinal autophagy by modulating the mTOR/Beclin1 pathway and decreasing Beclin-1 and LC3B levels (80). Total flavones of Abelmoschus manihot activate autophagy via NF-κB inhibition, increased LC3-II/LC3-I ratio and Beclin-1 ratio, and decreased p62 expression, to attenuate macrophage-mediated inflammation (97). The modulation of autophagy by TCM illustrates a sophisticated mechanism to maintain cellular homeostasis, clear intracellular pathogens, and regulate immune responses, thereby addressing a core pathogenic pathway genetically linked to CD. The emerging role of TCM in inhibiting ferroptosis opens a novel therapeutic avenue for protecting intestinal epithelial cells from this unique, iron-driven form of destruction, further expanding its repertoire in regulating cell fate in CD.

### 3.5 Antioxidant mechanisms

It is worthwhile emphasizing that oxidative stress represents another pivotal pathological component in CD pathogenesis. Studies have inferred that mucosal levels of reactive oxygen species (ROS) are markedly elevated in CD patients, primarily originating from infiltrating inflammatory cells and a dysfunctional mitochondrial electron-transport chain (142). Excessive ROS directly injures IECs, compromises barrier integrity, and activates multiple inflammatory signaling cascades, establishing a vicious cycle in which oxidative stress and chronic inflammation mutually exacerbate each other (143). Simultaneously, the endogenous antioxidant defense system is impaired, further aggravating oxidative damage (144).

Oleic acid markedly decreases colonic ROS and malondialdehyde (MDA) levels, increases glutathione (GSH) and superoxide dismutase (SOD) levels, exerts antioxidant effects, and improves intestinal inflammation (58). A previous study outlined that Xue-Jie-San potentially enhances Nrf2/GPX4-mediated anti-lipid-peroxidation by suppressing Notch1 signaling, thereby inhibiting mucosal lipid peroxidation and inflammation and alleviating colitis in CD rats (81). Qinghua Xiaoyong Formula confers antioxidant effects, ameliorates CD-like symptoms in mice, by inhibiting the PI3K/AKT pathway and increasing SOD, GSH, and MDA levels (84). Changyanning tablets markedly inhibit the formation of colonic ROS and lipid-peroxidation products (MDA), increase the levels of the antioxidant molecule GSH, restore redox homeostasis, and attenuate oxidative tissue injury (85). Peiminine enhances the activity of antioxidant enzymes (GSH-Px) by modulating the Nrf2/HO-1 pathway, thereby mitigating oxidative-stress-induced barrier injury (114). By counteracting oxidative stress, TCM agents disrupt a key driver of cellular injury and inflammation, contributing to the overall stabilization of the intestinal microenvironment in CD.

### 3.6 Enhancing immune responses and clinical efficacy

Traditional Chinese medicines can synergize with Western drugs to enhance clinical efficacy by modulating immune balance and inducing appropriate immune responses. The combination of Huangqin Tang granules with sulfasalazine corrects Th17/Treg imbalance, suppresses inflammation, enhances clinical efficacy, and reduces the risk of relapse (145). Chaihu Guizhi decoction significantly improves CD clinical outcomes, potentially by decreasing CD3, CD4, and the CD4/CD8 ratio and increasing CD8 to restore immune homeostasis (146). Chaihu Shugan Powder, in conjunction with Shenling Baizhu Powder, elevates Th17 and Treg levels, enhances immunity, alleviates inflammation, and suppresses inflammatory responses in CD patients (147). Meanwhile, the combination of modified Tao-Hua-Tang with Bu-Zhong-Yi-Qi-Tang inhibits the expression of IFN-γ, TNF-α, IL-2, and IL-17 in CD patients with spleen-stomach deficiency-cold syndrome during active phases, thereby controlling disease activity and attenuating disease severity and inflammation (148). Modified Lizhong Decoction combined with Western drugs decreases the Th17/Treg ratio, suppresses inflammatory responses, enhances immune function, and relieves CD symptoms (149). Fuzheng Huoxue Decoction lowers TNF-α levels, increases IL-10 levels, reduces the Th1/Th2 ratio, and alleviates CD (150). The combination of Buzhong Yiqi Decoction with infliximab decreases CD3^+^ and CD4^+^ levels, increases CD4^+^CD25^+^ Treg levels, modulates immunity, suppresses inflammation, and relieves symptoms in active CD patients with spleen-qi deficiency (151). Shenling Baizhu Powder combined with mesalazine enteric-coated tablets decreases CD4^+^ T lymphocyte count, increases CD8^+^ T lymphocyte count, decreases the levels of inflammatory cytokines, enhances immunity, improves clinical efficacy, reduces relapse rates, and delays disease progression in mild-to-moderate CD patients with spleen-deficiency and dampness-excess syndrome (152). Modified Banxia Xiexin Decoction combined with vedolizumab modulates the gut microbiota, induces immune responses, attenuates inflammatory injury, and exerts synergistic efficacy in moderate-to-severe active CD patients with mixed cold-heat syndrome (153). These clinical integrations demonstrate that TCM can act synergistically with Western biologics and drugs to achieve superior outcomes, potentially by providing a more comprehensive immunomodulatory background that enhances efficacy and promotes long-term remission.

### 3.7 Other mechanisms

Baizhu Fuling Decoction decreases VIP, VIPR1, and substance P via TLR4/NF-κB signaling, modulates neuropeptide secretion and receptor activity, and influences postsynaptic signaling, thereby regulating immune-cell function (70, 154). Huangqijiegeng Decoction and Huangqihuanglian Decoction both reduce VIP and TGF-β levels in the lung, colon, and serum of CD rats, improve vascular function, and promote repair of intestinal mucosal injury (155, 156). Earlier studies have identified that colonic glutamine (Gln) levels are significantly lower in TNBS-induced CD rats compared to controls, indicative of mucosal cellular injury. Besides, Kuijiekang restores mucosal Gln concentrations (74). Conversely, Baizhu Fuling Decoction alone or combined with Xianglian Wan lowers Gln levels, an effect interpreted as Gln, as a functional nutrient, being heavily consumed during inflammatory stress for tissue repair and immune responses, and returning to normal levels once inflammation is controlled (67). Quercitrin inhibits iNOS/COX-2, suppresses excessive NO production, and ameliorates early mesenteric vascular hyporeactivity in acute TNBS colitis (96). Loureirin B reverses epithelial-to-mesenchymal transition by inhibiting the IL-6/STAT3/NF-κB signaling pathway, down-regulating the expression of Snail, N-cadherin, and vimentin, and up-regulating that of E-cadherin (101). Daikenchuto selectively improves microcirculation in the ischemic colon by modulating the adrenomedullin/calcitonin gene-related peptide system, thereby alleviating inflammation and fibrosis (157). These diverse mechanisms underscore the holistic nature of TCM intervention, which extends beyond conventional anti-inflammatory and immunomodulatory approaches to improve the overall pathophysiological milieu of the gut, including neural, vascular, and metabolic aspects.

## 4 Discussion

This review systematically delineates the multifaceted mechanisms through which traditional Chinese medicine (TCM) alleviates the pathological progression of Crohn’s disease (CD). Cumulatively, the evidence indicates that TCM does not act via a single linear target, but rather through coordinated, network-level interventions that encompass immune modulation, inflammatory suppression, intestinal barrier reinforcement, and regulation of programmed cell death. This polypharmacological profile distinguishes TCM from conventional Western therapies and aligns well with the multifactorial nature of CD. The central finding of this review is that TCM acts synergistically through multi-target and multi-pathway mechanisms: it concurrently modulates immune cell polarization (Th1/Th2, Th17/Treg, M1/M2), suppresses multiple inflammatory signaling cascades (NF-κB, MAPK, PI3K/AKT, JAK/STAT), protects intestinal epithelial cells from diverse forms of cell death (apoptosis, pyroptosis, ferroptosis, and autophagic cell death), reinforces the physical barrier through tight junction proteins, and restores microbial homeostasis. This systemic regulatory paradigm fully embodies the holistic therapeutic philosophy intrinsic to TCM.

CD differs fundamentally from many autoimmune or inflammatory conditions in the architecture of its pathogenic network. Rheumatoid arthritis, for example, is sustained primarily by autoantibody-driven mechanisms; atopic dermatitis centers on epithelial barrier defects. CD, by contrast, appears to propagate through a self-amplifying loop. Genetic variants in NOD2, ATG16L1, and IRGM impair Paneth cell autophagy, weakening the intestinal barrier and permitting bacterial translocation. The resulting microbial signals then activate Th1/Th17 responses that inflict further epithelial damage, perpetuating a cycle of inflammation, cell death, and progressive dysbiosis (9, 10, 12, 24, 26). Because no single factor dominates this loop, interventions that target multiple nodes simultaneously may be necessary to interrupt the disease process.

Within the six pathogenic domains we have outlined, immune dysregulation and intestinal barrier dysfunction likely occupy a central position. Their primacy does not necessarily reflect temporal precedence; rather, most other pathogenic factors appear to channel their effects through these two processes. Genetic susceptibility alleles in NOD2, ATG16L1, and IRGM compromise barrier integrity largely by disrupting autophagy and Paneth cell function (10, 12). Environmental exposures—smoking, dietary patterns, and antibiotic use—together with gut dysbiosis, exert their influence mainly by modulating immune responses or disrupting the epithelial barrier (16, 18, 43). Programmed cell death, encompassing apoptosis, pyroptosis, and ferroptosis, is predominantly a downstream consequence of immune-mediated injury, yet it can amplify the inflammatory response (28, 35). This places the immune–barrier axis at the center of the disease process. Given this architecture, TCM multi-component formulas may offer a practical advantage over single-target biologics: rather than attempting to block one bottleneck, they can simultaneously modulate the immune–barrier axis that sits at the heart of CD pathogenesis.

Throughout this review, we observed that the multi-target regulation of CD by TCM is particularly exemplified by the integration and cross-talk among the NF-κB, PI3K/AKT, and Nrf2 pathways. NF-κB serves as a critical hub connecting immune dysregulation, inflammatory cascades, and intestinal epithelial cell apoptosis. Inhibitors of this pathway—such as berberine (52), baicalein (95), and α-cyperone (107)—simultaneously suppress Th1/Th17 polarization, reduce pro-inflammatory cytokine production, and preserve tight junction integrity. The PI3K/AKT signaling pathway participates in both inflammatory transduction and cell survival; its inhibitors, including clematichinenoside AR (53), pachymic acid (103), and magnolin (115), exert combined anti-inflammatory, anti-apoptotic, and barrier-protective effects. Recent evidence further supports this by demonstrating that Lactiflorin ameliorates experimental colitis through PI3K/AKT pathway modulation (158). Activation of the Nrf2 pathway inhibits ferroptosis through upregulation of GPX4, while concurrently mitigating pyroptosis-driven inflammation via NLRP3 suppression, as demonstrated by studies on peiminine (114), sclareol (113), and Xue-Jie-San (80, 81). These observations indicate that the action of TCM on CD is not confined to linear, single-pathway intervention, but rather represents an integrated modulation of the pathophysiological network, which aligns with the holistic philosophy inherent in TCM theory.

The therapeutic philosophy of TCM in the management of Crohn’s disease differs substantially from that of conventional Western medications. Current mainstream Western therapies predominantly employ monoclonal antibodies that target single factors such as TNF-α, IL-12/23, or integrins. Although these agents achieve considerable efficacy in a subset of patients, they carry inherent limitations. The inflammatory response in CD involves a complex network; when a single pathway is blocked, compensatory mechanisms may arise through alternative routes, leading to primary non-response or secondary loss of response in some patients (159, 160). In contrast, TCM—particularly in the form of compound formulas—exerts broader therapeutic actions. Rather than aiming for a single high-affinity target, it acts through synergistic interactions among multiple constituents and pathways, converging ultimately on the core pathological processes of the disease. This systems-level regulatory approach resonates with the current concept of “polypharmacology”—for complex diseases such as CD, multi-target intervention may hold advantages over the precision strike of a single agent.

From a clinical perspective, TCM offers complementary value to biologic therapies. In terms of safety, appropriately formulated TCM preparations are associated with fewer adverse effects and lower risks of immunogenicity and infusion reactions. In terms of convenience, oral or enema administration obviates the need for specialized facilities for intravenous or subcutaneous injection, rendering it more suitable for outpatient and home-based management.

Clinical evidence supports such combined use. TCM integrated with standard Western drugs has demonstrated enhanced therapeutic efficacy and reduced relapse rates across multiple studies (161). Several TCM formulas provide concrete examples of how multi-target agents address interconnected pathological processes. Shenling Baizhu Powder rebalances Th17/Treg differentiation while upregulating tight junction proteins (Occludin, ZO-1, E-cadherin) (76, 127), thus acting on both immune regulation and barrier repair. Xiangsha Liujunzi Wan suppresses PI3K-AKT and MAPK signaling while upregulating ZO-1 and Occludin (86). Xue-Jie-San modulates pyroptosis via the mTOR/Beclin1 pathway (80), ferroptosis through Notch1/Nrf2/GPX4 signaling (81), and inflammation through the TLR4/MyD88/NF-κB axis (82). The concurrent modulation of immune balance, barrier integrity, and cell death pathways across these formulas represents an advantage that single-target biologics do not offer. For instance, although infliximab is effective in CD, a substantial proportion of patients fail to respond initially or lose response over time, often due to immunogenicity or compensatory activation of other inflammatory pathways (158, 160). In contrast, the multi-pathway profiles of these TCM formulas may circumvent such compensatory escape by targeting multiple nodes at once, potentially offering a more durable therapeutic strategy. Given that sustained remission remains a challenging goal in CD management, TCM may offer additional benefits in this regard. In summary, TCM is not intended to replace conventional therapy, but rather serves as a complementary strategy that addresses the complex pathophysiology of CD more comprehensively through multi-target modulation.

Despite the encouraging evidence currently available, research on TCM for Crohn’s disease still faces critical challenges that must be addressed to facilitate its rational integration into clinical practice. It should be acknowledged that a considerable proportion of the experimental evidence summarized in this review originates from studies published in Chinese-language journals or from lower-tier international journals, and many of these studies lack rigorous methodological features—such as sham controls, blinded outcome assessment, independent replication, or adequate sample size calculation. While these investigations provide valuable preliminary insights into the therapeutic mechanisms of TCM in CD, their methodological limitations preclude definitive conclusions and underscore the urgent need for well-designed, high-quality preclinical and clinical studies to validate and extend the current findings. Moreover, the descriptive nature of the available literature—largely reporting positive results without systematic consideration of negative or inconclusive findings—may introduce publication bias. Future research should prioritize transparent reporting and independent validation to strengthen the evidence base for TCM in CD management.

First, mechanistic elucidation needs to be deepened. The available evidence is derived predominantly from conventional biochemical approaches. Emerging technologies such as network pharmacology, single-cell sequencing, and spatial transcriptomics offer more systematic means to dissect the interactions between TCM constituents and their targets.

Second, the issue of standardization remains unresolved. The compositional complexity of TCM formulas and variability in manufacturing processes limit reproducibility. Prioritizing the development of analytical methods such as HPLC fingerprinting and mass spectrometry to ensure batch-to-batch consistency is of critical importance.

Third, the quality of clinical research requires improvement. More large-scale, multicenter, randomized double-blind trials employing objective endpoints—such as the Crohn’s Disease Activity Index (CDAI) and fecal calprotectin—are needed to evaluate efficacy. Stratifying patients according to TCM syndrome patterns is recommended to validate the advantages of individualized therapy; this is both a hallmark of TCM and an area in which clinical verification remains insufficient.

Finally, precision-oriented approaches and combination strategies hold promise. Patient selection based on immune profiles, genetic or microbial biomarkers, and rational integration of TCM with biologics are all directions worth exploring.

In summary, substantial progress has been made in mechanistic research on TCM for CD. However, translating these findings into clinical practice will require multidisciplinary collaboration across basic science, analytical chemistry, clinical trial design, and regulatory science. Through systematic investigation, TCM has the potential to evolve from an experience-based practice into an evidence-based therapeutic modality with well-defined mechanisms and robust supporting data, thereby offering broader options for patients with CD.

## 5 Conclusions and future perspectives

In summary, this review provides a comprehensive mechanistic framework positioning TCM as a multi-target therapeutic system for CD, acting through immune modulation, inflammatory suppression, barrier protection, and regulation of cell death pathways. Despite substantial progress, challenges remain in mechanistic depth, product standardization, and clinical trial quality. Future efforts should integrate network pharmacology, single-cell technologies, and syndrome-stratified randomized trials to transform TCM from an experience-based practice into an evidence-based modality. With multidisciplinary collaboration, TCM holds promise to offer CD patients broader and more personalized therapeutic options.
